# Nanotechnology as a Versatile Tool for ^19^F-MRI Agent’s Formulation: A Glimpse into the Use of Perfluorinated and Fluorinated Compounds in Nanoparticles

**DOI:** 10.3390/pharmaceutics14020382

**Published:** 2022-02-09

**Authors:** Joice Maria Joseph, Maria Rosa Gigliobianco, Bita Mahdavi Firouzabadi, Roberta Censi, Piera Di Martino

**Affiliations:** 1 School of Pharmacy, University of Camerino, 62032 Camerino, Italy; joice.mariajoseph@unicam.it (J.M.J.); bita.mahdavi@unicam.it (B.M.F.); piera.dimartino@unicam.it (P.D.M.); 2 Percuros B.V., 2333 Leiden, The Netherlands; m.gigliobianco@percuros.nl; 3 Dipartimento di Farmacia, Università “G. D’Annunzio” Chieti e Pescara, 66100 Chieti, Italy

**Keywords:** magnetic resonance imaging, perfluorocarbons, imaging agent, nanosystems, nanoparticles, fluorine

## Abstract

Simultaneously being a non-radiative and non-invasive technique makes magnetic resonance imaging (MRI) one of the highly sought imaging techniques for the early diagnosis and treatment of diseases. Despite more than four decades of research on finding a suitable imaging agent from fluorine for clinical applications, it still lingers as a challenge to get the regulatory approval compared to its hydrogen counterpart. The pertinent hurdle is the simultaneous intrinsic hydrophobicity and lipophobicity of fluorine and its derivatives that make them insoluble in any liquids, strongly limiting their application in areas such as targeted delivery. A blossoming technique to circumvent the unfavorable physicochemical characteristics of perfluorocarbon compounds (PFCs) and guarantee a high local concentration of fluorine in the desired body part is to encapsulate them in nanosystems. In this review, we will be emphasizing different types of nanocarrier systems studied to encapsulate various PFCs and fluorinated compounds, headway to be applied as a contrast agent (CA) in fluorine-19 MRI (^19^F MRI). We would also scrutinize, especially from studies over the last decade, the different types of PFCs and their specific applications and limitations concerning the nanoparticle (NP) system used to encapsulate them. A critical evaluation for future opportunities would be speculated.

## 1. Introduction

For an early stage detection of a disease or a routine medical checkup, it is preferable to have a non-invasive, cost-effective, and patient-friendly technique that makes it approachable and reassuring [1]. In vivo molecular imaging is one such technique that can visualize, quantify, and characterize biological processes at the cellular and molecular levels in a living entity for pretreatment planning, prognostics, and post-treatment surveillance. Screening (detecting diseases in early stages) and identifying the extent of the disease, monitoring for disease recurrence, personalized medicine (selecting patient and disease-specific therapeutic treatment), measuring molecular-specific effects of treatment, predicting and monitoring response to therapy are within the realm of its possibilities [2]. The parameters taken into consideration before choosing an imaging modality are its depth of penetration, temporal resolution (how quickly the image can be acquired/acquisition time), anatomical resolution, spatial resolution, sensitivity (ability to depict molecular features of imaging areas), multiplexing capabilities (ability to simultaneously image/visualize multiple molecular targets), etc. [3]. In most cases, the imaging modality requires the presence of an entity called a contrast agent (CA) to enhance the distinction between the target tissue and the background that overcome the issue of low sensitivity and hence obtain a good quality image. Some of the modalities for molecular imaging are optical, photoacoustic (PAI), ultrasound (US), computed tomography (CT), positron emission tomography (PET)/single-photon emission computed tomography (SPECT), X-ray, and MRI.

## 2. Types of Molecular Imaging

In optical imaging, the near-infrared (NIR) and visible part of the optical spectrum is feasible for detection with the help of fluorochromes [4]. Fluorochromes injected into the bloodstream emit wavelengths up to about 700 nm. Microscopic lenses are utilized throughout the near infrared spectroscopy and imaging (NIRS) ranging from 600 to 900 nm, with near-transparency of living tissue. Two modes of optical imaging are fluorescence and bioluminescence imaging [5]. Higher intensities (above approximately 1200 W/cm^2^) overheat the tissues, preventing deeper penetration by upheaving the light intensity used [4]. Optical imaging is an inexpensive technique with temporal resolution in minutes and spatial resolution in millimeters (mm) and is well-suited for diagnostic and microscopic studies of cells and tissue sections in real-time scans. However, the penetration depth, which is limited to a few millimeters, autofluorescence, and poor spatial resolution at greater depths limits the present applicability of optical molecular imaging in clinical practice [6,7].

Photoacoustic imaging (PAI), also known as optoacoustic or thermoacoustic imaging, is a modality for non-invasive visualization based on converting laser into heat [8]. Known for good penetration depth (mm–cm), it can image semi-transparent objects, soft biological tissue, and biological samples. Imaging agents are frequently used, including methylene blue, gold NPs, etc. that have a superior ability to absorb light to produce vivid photoacoustic images [9]. The technique still suffers from certain technical hurdles like the indispensable coupling of the instrument to the subject and the possibility to image only soft tissues and not bones or air structure, and due to the moderate laser energy, a small part of the body can only be imaged at a time.

Ultrasound (US) is a rapid, real-time, soft-tissue imaging technique that is rather inexpensive [10]. However, the spatial resolution (mm–cm) is inconsistent depending upon the required penetration of depth, and it is unsuitable for adult brain imaging as it does not penetrate air gaps or bone. Currently, US is used in the clinic and has an excellent sensitivity [11]. Unsuitable for multiplexing, and its imaging is limited to soft tissues with the unavoidable physical coupling of the device to the subject.

Computed tomography (CT), positron emission tomography (PET)/single-photon emission computed tomography (SPECT) imaging, often used in sequence, uses ionizing radiation. Notwithstanding the use of radiations, CT is the most commonly used clinical imaging modality with its advantage of limitless depth penetration. CT provides mm–cm resolution and a good contrast between hard and soft tissues with a typical scan taking up to 3–4 min to acquire [12]. CT provides mainly anatomical information but has poor sensitivity, specificity, and temporal resolution [13]. PET/SPECT is a radionuclide molecular imaging technique that allows for whole-body imaging of molecular targets or processes, has 1–2 mm resolution, and typically has scan time in minutes. Yet, the need for freshly prepared radioactive chemicals makes it a costly and complex technique. Even though this technique has excellent sensitivity, it has a poor spatial resolution [7,14,15]. PET/SPECT has the great advantage of identifying diseases at early stages since it visualizes molecular targets affected by changes at an earlier stage than that occurring in structural tissue.

The state-of-the-art X-ray imaging uses an X-ray source to get the images and have an inherent high spatial resolution. The instrumentation and use of it are relatively inexpensive, though the imaging process should be precisely monitored. The absorption of X-rays is directly proportional to the atomic number of the absorptive element [16]. A contrast medium is used elsewise soft tissues will not be visible in the image. Except for using an ionizing radiation source like X-rays which can cause radioactive damages in the human body if exceeded a safe dosage, it is essentially a very economic diagnostic technique with a straightforward image acquisition [4]. Refer to the review by Gambhir et al. [3] and Debbage et al. [4], for further explanations on each of the previous techniques.

MRI is an extremely versatile anatomical, functional, and diagnostic imaging technique which excels at deep soft tissue imaging and provides disease information [17]. It can stipulate finer distinctions between soft tissues at higher resolution (mm range) than the previously mentioned imaging techniques without the need for ionizing radiation [18]. In 2010, the Food and Drug Administration (FDA) center for devices and radiological health started an initiative to reduce unnecessary radiation exposure from medical imaging [19], favoring the use of techniques that do not require the use of radioactive sources. Compared to other imaging techniques, limitless sample penetration, the possibility to manipulate contrast between tissues of interest by altering the scan acquisition parameters, and a better differentiation among fat, water, muscle, and other soft tissues make MRI one of the most sought imaging techniques despite low sensitivity (including coil sensitivity), lower temporal resolution (scan time depends on the required resolution and the field of view size) and a time-consuming data acquisition process [3]. Its safety profile allows repetitive imaging sessions, an exigent aspect for prolonged, chronic disorders [20].

Since every imaging technique has its unique benefits and drawbacks, combining imaging modalities (multimodal imaging) can offer synergistic advantages over a single modality to compensate for each imaging method’s inherent limitations, subsequently to obtain more accurate and informative images. In fact, in most studies, multimodal imaging has become a trend both in research and clinic applications for meticulous examinations [21,22,23]. Table 1 compares the different parameters of all the imaging modalities discussed including optical imaging, PAI, US, CT, PET, SPECT, X-ray, MRI.

## 3. Principles of NMR and MRI

Depending on the appropriately tuned amplifiers and transceiver coils, in theory, any nuclear magnetic resonance (NMR) active nucleus can be used for imaging by MRI [24]. A nucleus with a spin quantum number of ½ (e.g., ^1^H, ^3^He, ^13^C, ^14^N, ^15^N, ^19^F, ^19^O, ^31^P, etc.) is designated to be in two spin states and the direction of spin alignment depends on the sign (+/−) of the gyromagnetic ratio, one of the two spin states will align along the magnetic field (ground state, lower energy), whereas the other one will align against it (excited state, higher energy). When an external magnetic field is applied, the spins in the ground state can be promoted to the excited state after absorbing the energy [25]. Upon the termination of the external magnetic field, the spin returns to its equilibrium state (ground state) by a process called relaxation. There are two processes involved, each with an exponential time constant (*Ti*, *i* = 1,2): ‘*T*_1_’ (longitudinal or spin-lattice) or ‘*T*_2_’ (transverse or spin-spin) relaxation times [26]. These parameters help in determining the signal/contrast-to-noise ratio (SNR) and the image resolution.

The distinctiveness in the color density of the images of the biological tissue obtained in the MRI (which is the contrast) is fundamentally due to the difference in the rate of relaxations of the nucleus under study. Standard proton MRI (^1^H MRI) imaging relies upon the detection of differences in relaxation of water protons to their ground state (relaxation rates) among tissue types, whose signal strengths are reconstructed to give a well-defined distinctive final image [27]. While conventional MRI does not necessarily require the addition of an external CA, there are circumstances when there is not sufficient difference in the relaxation rates of protons among the tissue types (bones, bodily fluids (soft tissues), fat, etc.) to produce a decent contrast. In such cases, an external CA is administered to alter the endogenous proton relaxation times (*T*_1_/*T*_2_) to obtain highly enhanced tissue contrast signals. Gadolinium(III)-based CAs (GBCA) are among the widely used examples of inorganic substances used for ^1^H MRI. Currently, a few others are also being explored as potential MRI CAs, including perfluorocarbon (PFC) compounds and fluorinated molecules, which will be extensively considered in this review.

### 3.1. Gadolinium Based Contrast Agents (GBCAs)

GBCAs are paramagnetic coordination complexes comprising of a Gadolinium-III (Gd(III)/Gd^3+^) ion and a chelator that independently do not emit MR signals but can bring about a significant reduction of the *T*_1_ of nearby water protons [28]. Annually, millions of patients globally undergo MRI scans who receive GBCAs. The lanthanides like Gd are highly coveted CAs due to their intrinsic paramagnetic properties, favorable relaxation time, [29], and stable shelf life. GBCAs permit the imaging of tissues that are less sensitive to motion (hence better quality images) and higher throughput by shortening *T*_1_ of the proton [28]. The contrast enhancement function comes from Gd^3+^ that has seven unpaired electrons. After administering the CA, the diagnostic image is procured while the patient is in the scanner. Generally, the diagnostic and prognostic information attained from MRI predominates the information given from other techniques. Several GBCAs have gained regulatory approvals, including Eovist^®^ (gadoxetate disodium), Omniscan^®^ (a gadodiamide), Gadavist^®^ (gadobutrol), Optimark^®^ (gadoversetamide), etc. [30,31]. The free Gd^3+^ ion is toxic since its ionic radius is relatively close to zinc, calcium, or iron [32]. Likely interference with calcium ion channels in the living entity is plausible. Gd^3+^, therefore, needs to be cocooned within chelator (most often used is organic ligands) to avoid those toxicity issues [33,34]. Two classes of chelates developed to complex Gd: linear or macrocyclic organic ligands evade the release of free Gd^3+^ and make the resulting complexes kinetically and thermodynamically stable [35].

However, in 2006, GBCAs were associated with a devastating and potentially fatal condition called nephrogenic systemic fibrosis [36], recurrently reported in patients suffering from renal deficiency, and its onset can occur months after the last GBCA administration [28]. Furthermore, it is prevailing that some fraction of the residual Gd^3+^ can remain in the body for long periods, although the chemical form or its whole-body distribution is still obscure [28]. Because of the low sensitivity of MRI, formulation stipulates a high concentration of Gd^3+^, typically 0.1 mmol kg^−1^ body weight (approximately 0.5 M aqueous solution) that is hypertonic relative to body fluids [37]. Notwithstanding this, some macrocyclic GBCAs are still sanctioned and can be administered to the patients but in the lowest possible doses. Together, these conclusions have led to renewed interest in finding alternatives to using Gd^3+^ for MR contrast [38,39]. Further, in 2017, the European medicines agency (EMA) and FDA confirmed the necessity of restricting the use of some linear GBCAs because they tend to release Gd ions in the biological environment [40,41]. For a deeper understanding of GBCAs, the reader is referred to the following reviews [27,28,34,39].

### 3.2. Fluorine as a Contrast Agent

There is variation among different elements of an NMR active nucleus for their relative natural abundance and response to a magnetic field, meaning that the NMR signal per mole of the compound varies from element to element [24]. Choosing an imaging nucleus from the different NMR active elements depends on its properties entailing to its inherent physical, chemical, and biological properties. In 1977, shortly after the invention of ^1^H MRI, Holland et al. [42], have demonstrated the feasibility of fluorine-19 suited for fluorine-MRI (^19^F MRI), which paved the way for new research avenues in molecular and cellular imaging. ^19^F MRI is anticipated to be a promising imaging tool in the future due to unambiguous detection, acceptable in vivo acquisition times, and relatively high spatial resolution [43]. The external addition of a suitable fluorinated compound (also called a probe/tracer/label) is a prerequisite for ^19^F MRI/magnetic resonance spectroscopy (MRS).

Only insignificant amounts of endogenous fluorine are embedded in the teeth and bone matrix of the human body. This immobilized fluorine (<10^–6^ M) has only a very short *T*_2_ relaxation as they are in the solid-state and cannot be detected by ^19^F MRI (that is below the detection limit), which extinguishes the possibility of intrinsic background signals, implying potentially high SNR [44]. Using the same scanner and the receiver electronics of ^1^HMRI with retuned radiofrequency coils/dual-tuned coils, ^19^F-images can be superimposed on anatomical, high-resolution ^1^H images, generating hotspot ^19^F-images (hybrid ^1^H/^19^F MRI) [45,46,47]. The MR effect of the additional element (^19^F here) does not disturb the local magnetic field either and adds a second colored layer of complementary information to the corresponding grayscale ^1^H image, hence called “hot spot” [48,49]. Aside from that, ^19^F is a natural halogen, non-radioactive stable isotope of fluorine [50], unlike the radioactive isotope ^18^F used in PET imaging [51], and thus it is not necessary to have advanced synthetic skills to introduce fluorine into a probe.

### 3.3. Similarity between Fluorine and Hydrogen

^19^F exhibits the NMR phenomenon like ^1^H, which has one unpaired proton and no unpaired neutrons, and thus with a net spin of ½. Many fluorinated compounds that are non-toxic and chemically inert provide a non-invasive means to study biological systems. When an NMR-active nucleus is placed in an external magnetic field of strength *B*, it can absorb a photon of frequency *ν* that depends on the gyromagnetic ratio (*γ)* of the particle.

(1) ν=γ B

In Equation (1), *B* is the strength of the applied magnetic field (in Tesla [*T*]), and *γ* is the gyromagnetic ratio of the nucleus (in *MHzT*^−1^). The similarity of ^19^F’s gyromagnetic ratio to ^1^H is another strong suit that makes ^19^F the second most sensitive stable nuclei for MRI followed by ^1^H (Table 1) [52,53]. At 3*T*, the typical field strength for clinical MRI scanners, *ν* is 128 MHz for ^1^H and 120 MHz for ^19^F [37]. These frequencies (commonly known as ‘resonance frequencies’) lie in the radiofrequency (RF) range, and hence, MRI signals are RF signals. ^19^F resonates at a resonant frequency of 94% that of ^1^H [54]. A huge advantage of MRI over other imaging methods is that RF pulse is non-ionizing radiation and per se can penetrate deep into soft tissues [18]. Once the wave packet of frequency (in this case RF pulse) is applied, as already disclosed, the ground state spins obtain the energy to transition to the excited state, whose energy can be posited by Equation (2)

(2) E=h ν

where *h* is Planck’s constant (6.626 × 10^−34^ joules (J)-second (S)). Denoting the population of the ground state as *N*_G_ and that of the excited state as *N*_E_, the MR signal intensity is proportional to the population excess between the two states that can be secured by Equation (3) [37]

(3) $$Population\;excess\;\left(Spin\;polarization\right)=\frac{\;\left(N_{G}-N_{E}\right)}{\left(N_{G}+N_{E}\right)}$$

At thermal equilibrium, the distribution of spins between the two states obeys Boltzmann’s law. The population ratio, which is the ratio between the spins in the excited state to the ground state, (*N_E_*/*N_G_*), is obtained by Equation (4) which is 0.9999802 for ^1^H and 0.9999814 for ^19^F [37].

(4) $$\frac{N_{E}}{N_{G}\;}=e^{\frac{-\Delta \epsilon }{kT}}$$

where ∆*ϵ* is the energy difference between the excited and ground state, *k* the Boltzmann constant (1.381 × 10^−23^ JK^−1^), and *T*, the absolute temperature in kelvin (K). Hence, the MR signal is the output of a tiny population difference between the two states as only 9–10 spins out of almost 10 lakhs contribute to the sequel. It sums to the fact that in the absence of CAs, MRI is an intrinsically low-sensitive technique. NMR receptivity is the absolute NMR sensitivity of a nucleus at its natural abundance [24]. ^1^H has the most distinguished receptivity of any nucleus. To identify an absolute value of receptivity for other nuclei, it is represented relative to ^1^H, with ^1^H having a receptivity of 1. ^19^F atom with a natural abundance of 100%, has a receptivity of 0.834 relative to ^1^H, and the fact that it is not a particularly rare (or expensive) element [52] makes it exemplary suitor for replacing ^1^H. It has a relative sensitivity of 83% compared to ^1^H and is essentially devoid in biological tissues [52]. Table 2 compares the properties of hydrogen and fluorine that present a large extent of similarity between them except for the chemical shift, for which fluorine is electron-rich, so possesses a high chemical shift.

## 4. Perfluorocarbons (PFCs) as Contrast Agents for ^19^F MRI

### 4.1. Physicochemical and Biological Properties of Perfluorocarbon (PFC) Molecules

The signal from ^1^HMRI originates from nearly two-thirds of all protons present in the body, and for ^19^F MRI to produce an equivalent image, a very high density of ^19^F nuclei needs to be comprised in the CA to reach an optimal concentration. One way around the prerequisite of high F-concentration is by using PFCs and their derivatives, where all the protons (H’s) of the hydrocarbon chain are switched to ^19^F nuclei [54,55,56,57]. Other options would be fluorine-rich macromolecules, nanosystems, and paramagnetic metal-containing agents. PFCs are one of the most biologically inert organic molecules ever known and have been under scrutiny for the last few decades [55]. Usually, fluorination enhances the bioavailability of the new drug by increasing lipophilicity [58]. Since fluorine is the highest electronegative element in the periodic table, the covalent C–F bonds are among the strongest known bonds that attribute to the high thermal, chemical, and oxidative stability [44,59].

In addition, they have higher compressibility, higher gas-dissolving capacities, extreme corrosive resistance, high density, high vapor pressure, high fluidity, low cohesive forces, lower dielectric constants, low refractive indices, low polarity, weak intermolecular interactions, and lower surface tension [44,57,60]. The high density and compressibility enable PFCs to reduce the contact force. Even at very high in vivo doses, this class of molecules is biologically compatible with no toxicity partly because of their high hydrophobicity and significant lipophobicity that gives them the tendency to segregate from the surrounding environments [55,60,61].

**Figure 1:**
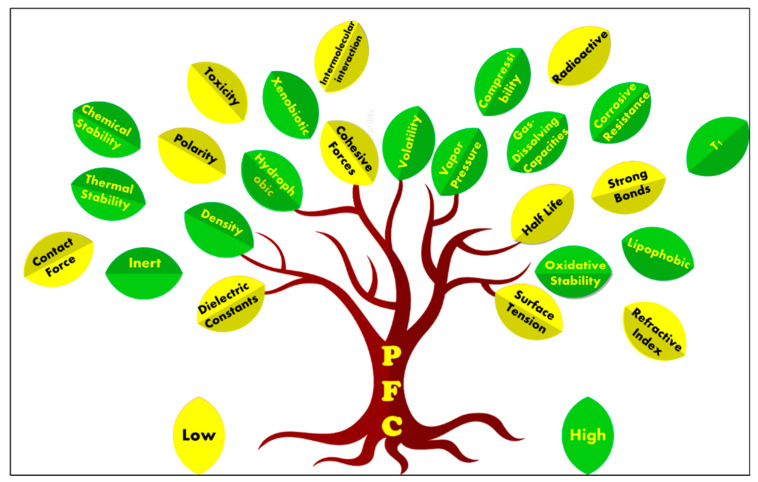
The unique properties of perflurocarbon compounds represented by a tree and its leaves. The green leaves represent the properties of PFC in which the values are higher, and the yellow leaves represent lower values.

Between the degree of hydrophobicity and lipophobicity, the former outstands the latter [62,63]. Furthermore, they are xenobiotic, there are no known enzymes that metabolize PFCs in vivo [62], and are degradation resistant [50] at typical lysosomal pH values or in the volatile form such as Freon^®^. Furthermore, most PFCs, within the molecular weight range of 460–520 Da, exhibit no significant toxicological risks, carcinogenicity, teratogenicity, or mutagenicity [53,54]. The notable properties of PFCs are represented graphically as a tree and its leaves in Figure 1.

### 4.2. PFC Molecules in a Nanoparticle Formulation

The bottleneck factor for manipulation of most PFCs, bearing in mind its high fluorinated nature, is their simultaneously hydrophobic and lipophobic character, which makes them, in most cases, insoluble in any medium [61,64]. This peculiar feature has an ultimate implication for the design and formulation of MR imaging agents. One way around is to encapsulate/hide PFCs inside a biocompatible coating or capsule to optimize their biopharmaceutical properties. These formulations are accomplished by making nanoemulsions or microemulsions stabilized by surfactants whose employment might also influence cellular uptake [65]. Some of the frequently used surfactants are pluronics and phospholipids, the surface-active agents that can form a film around the dispersed globules of PFC by adsorbing at PFC–water interfaces and reducing the interfacial surface tension (water–PFC interfacial tension is around 60 mN/m) [54]. In many instances, one or more surfactants are blended to get the desired characteristics. By way of alternative, nanoscale micelle had been reported that can self-assemble without the need of surfactants in an aqueous solution like amphiphilic poly-fluorinated polymers [66,67]. It is a pragmatic choice since the emulsification process with a surfactant is adding yet another complexity to the system in addition to equipment and different reagents, and practically the outcome is the formation of a heterogeneous system with disparate NP size.

There are various preparation procedures reported for formulating a stable emulsion with longer shelf life. The techniques are identified as a top-down and bottom-up approach [68]. The commonly used methods are from the bottom-up approach, including emulsion-solvent evaporation method, double emulsion, and evaporation method, the emulsions-diffusion method using a homogenizer or a sonicator, nanoprecipitation (solvent displacement), salting out method, microfluidization, etc. [69]. A perfect nanoemulsion would depend on the definitive desired application, and it is always a balance of emulsion stability, the desired outcome, and body clearance. The therapeutic effect of the nanosystem can be further reduced after administration into the body by proteins adsorbed to the nanosystems surface by so-called ‘protein corona’ formation [70]. There are camouflaging ways to prolong the nanosystems circulation in the blood, including modification of its surface with polyethylene glycol (PEG) [71] shell, dextrose, polysaccharide (chitosan, hyaluronic acid, fucoidan), albumin, or zwitterion, etc. [72]. Research has found that PFC emulsions have no adverse renal toxicity [73], thereupon might be the best alternative for people with kidney complications.

The nanosystems can be chemically/physically modified with a targeting ligand (antibodies and peptides) to amplify their accumulation to the target site [74,75]. Compared to non-targeted nanosystems, targeted nanosystems seem to stay longer in the blood circulation. The former immediately accumulates in the liver and spleen post-injection. Active targeting involves conjugating the NPs with ligands that can specifically bind to cellular antigens in the pathological site of interest. On the other hand, a passive targeting strategy exploits the abnormalities of tumour vasculature that cause leakage of macromolecular agents and NPs into the tumour interstitium, the phenomenon known as the enhanced permeability and retention (EPR) effect [76]. Concomitantly it is possible to equip the nanosystems with other payloads (drugs, genes, protein, etc.) to craft them as a therapeutic agent. Likewise, a theragnostic agent could potentially combine an imaging and a therapeutic agent into a single formulation [77]. There are also ‘‘smart’’ systems that can respond to the biochemical or physiological abnormalities (pH, temperature, the concentration of ions or metabolites, hypoxia, enzyme, etc.) to modulate their SNR by their physical-chemical properties [78,79,80].

The primary clearance system of the majority of the nanosystems in humans is the immune system. The first line of defense that they encounter within the body is the reticuloendothelial system/mononuclear phagocyte system (RES), which can, later on, undergo opsonization (surface adsorption of serum proteins to the nanosystem), and phagocytosis (engulfing and destruction/removal of foreign materials from the bloodstream into organs like liver and spleen) [81]. In principle, the duration of PFCs remaining in the body/exact clearance depends on their chemical structure, individual intrinsic properties, the mode of administration (intravenously, orally, or inhalation), molecular weight, and vapor pressure/volatility [73]. The half-life of the PFCs has an inverse relation to their volatility, which can range from minutes to years. Due to the hydrophobicity of PFCs, they have slow diffusion in their natural form that can prolong their stay in the target site.

After being drawn up by RES, PFC emulsions are diffused back into the blood, where they dissolve in plasma lipids and are carried to the lungs to be expelled out mainly through exhalation by the lungs [56,73,82]. Even though PFC is intrinsically inert, there are reports of severe retention of PFCs in the liver, spleen, and lungs [83,84,85], and the effect of the PFC when stayed too long in vivo or their intracellular fate is currently undetermined [53,86]. In general, nanosystems of size less than 10 nm, exceedingly are devoured by the renal clearance system, 20 to 100 nm by far, is the optimal size range to avoid physiological barriers, 100 to 200 nm particles have extended potential for prolonged circulation, and size greater than 200 nm is retreated almost certainly to spleen and liver and has the possibility for capillary clogging [87]. Frequently, a formulation contrived by design considerations including droplet sizes ranging from 10 to 200 nm (to take advantage of passive targeting by EPR effect), a low polydispersity index (narrow size range) of less than 0.2, and a high fluorine concentration [88]. So far, reported, PFCs have a half-life for blood clearance ranging from 3 h (h) to 42 h and tissue half-life ranging from 4 to 8 days for PFOB, up to 65 days for PFTA, and over 100 days for PFCE [53,89,90].

### 4.3. Types of PFC Molecules

One of the critical aspects of fabricating an optimized ^19^F MRI CA using PFC is the chemical structure of the CA itself. The sensitivity of PFCs as MRI CA is highly dependent on the number of fluorine atoms present in the CA, and to increase the signal intensity, the number of fluorine atoms per imaging agent molecule is a vital parameter. In addition, the dosage, magnetic field strength, detector design, etc. affect the sensitivity. PFCs can be detected and quantified directly by ^19^F NMR, an excellent technique for preliminary studies in ^19^F MRI. One of the colossal benefits of using PFCs is that their ^19^FNMR has an extensive chemical shift range (~400 ppm), which asserts the marginal possibility of signal overlap when multiple agents are simultaneously studied [91].

PFCs are clear colorless dense liquids, and their molecular structures generally fall into several classes, including, aromatic–hexafluorobenzene (HFB) [92], trans-1,2-bis(perfluorbutyl)-ethylene (TBPE), 2,3,4,5,6-pentafluorostyrene (PFS)), saturated linear–perfluoro-tert-butanol (PFTB), perfluoropropane (PFP), perfluorohexane (PFH), perfluorononane (PFN), perfluorooctyl bromide (PFOB), perfluorooctanoic acid (PFOA)), saturated ring system–perfluorodecalin (PFD), perfluoro-1,3-dimethylcyclohexane (PFDCH), perfluoroperhydrophenanthrene (PFPHP), perfluoroethers and polyether–perfluoro-15-crown-5 ether (PFCE), perfluoro-2,2,2’,2’-tetramethyl-4,4’-bis(1,3-dioxolane) (PTBD) [93], fluorescent ‘blended’ PFPE amides (FBPA) [94], superfluorinated probe (PERFECTA), perfluoropolyether (PFPE), perfluoroamines–perfluorotriethylamine (PFTA), perfluorotributylamine (FC-43), ^19^F imaging tracer (^19^FIT) [95] and perflurosilanes -(pentafluorophenyl)triethoxysilane (PFPTS), ^1^H,^1^H,^2^H,^2^H-Perfluorooctyltriethoxysilane (PFOTS), Trichloro(^1^H,^1^H,^2^H,^2^H-perfluorooctyl)silane (TCPFOS) [65] (refer to Table 3 for detailed information on each of the PFCs mentioned). Depending on its structure, the same could be classified as cyclic, branched, linear, and non-linear. Currently, even though not all of the described PFCs are applied in ^19^F MRI, most of them hold the potential to be trialed for biomedical applications and then sieve them for clinical trials. There is a variety of PFC’s presently available, in which some of them are commercially used for applications such as an ultrasound probe or cell tracking agent.

1. Perfluorooctyl bromide (PFOB/perflubron) is one of the most used PFC materials in biomedicine [96,97]. It is a tasteless and odourless liquid and is extensively adapted for artificial oxygen carriers [73]. It is a dense liquid with a low diffusion coefficient inside the blood, has a longer blood circulation time, and excreted out faster than most other PFCs. It has a linear structure, low surface tension, high specific gravity, finite lipophilicity due to a covalent bond to bromine which enhances its clearance rates from the body [98]. PFCs with additional chemical elements, such as a bromine atom in PFOB, tend to have a short biologic half-life value [99]. They are scarcely absorbed in the gastrointestinal tract, wherefore could be ingested in large doses for bowel imaging [62]. Albeit PFOB displays multiple ^19^F peaks (eight peaks, one for each CF_n_ moiety) that compromises its sensitivity, it is possible to minimize undesired resonance peaks by including pre-saturation RF pulses with MRI pulse sequences before readout [98,100].
2. Perfluoropolyether (PFPE) is the simplest linear polymer that is an excellent ^19^F MRI probe as they provide a single sharp resonance for hassle-free identification, maximizing the SNR and eradicating any chemical shift artifact of the PFC [101]. This class of molecule is known for its remarkable thermal stability and high molecular mobility that improves ^19^F MRI sensitivity [102]. Linear PFPE possesses end groups susceptible to chemical modification by synthetic strategies [103,104] to bolster additional scope in multimodal imaging. This polymer has short *T*_1_ (600 ms) and adequately long *T*_2_ (160 ms), the desired trait for an imaging agent. They own a linear structure and high content of MR equivalent ^19^F nuclei per molecule, with >40 chemically equivalent fluorine [88] that should theoretically give them single resonance. The carbon-oxygen bonds stipulate an increased bond rotation that aids them to be better biodegradable [59].
3. Using macrocyclic perfluoropolyethers (PFPEs), e.g., the 12, 15, or 18 crown ethers with their high number of equivalent fluorine atoms (16, 20, and 24, respectively) assure an outstanding NMR performance, notably of chemical shift artifacts, SNR, single sharp resonance peak that enable for unambiguous identification, etc. Macrocyclic PFCs such as the perfluoro-15-crown-5 ether (PFCE) assure a substantial improvement in MRI sensitivity with 20 chemically equivalent fluorines (NMR resonance at around ~−92.5 ppm) [98] and is one of the most explored PFC [105,106,107,108,109,110,111,112].
4. PERFECTA (su*PERF*luorinat*E*d*C*ontras*T A*gent) has 36 chemically equivalent fluorine atoms per molecule, which gives them a single major resonance in FNMR [113]. Unlike other PFCs, they have a polar hydrocarbon core. They are found to have reliable cellular compatibility from the preliminary in vivo F-MRI experiments [113,114].

### 4.4. The Sine Qua Non of Fluorinating Agents in ^19^F MRI for Clinical Translation—Chemical, Physical and Biological Traits

**Figure 2:**
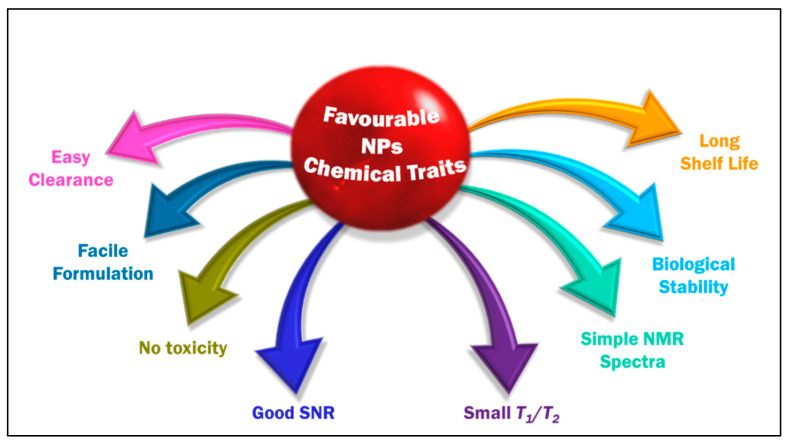
The favorable characteristics of a CA for ^19^F MRI.

Even after four decades into the research of ^19^F MRI, none of the PFC formulations have gained clinical approval [83]. Umpteen requisites should be actualized to extend a formulation into the clinic. Until August 2021, as many as six PFC ^19^F MRI agents (phase 1) are in clinical trials, mainly employing PFP for cell labelling and lung imaging [115]. There are several parameters and requirement norms for any CA formulation to be spanned to the clinic in ^19^F MRI. In a nutshell, some of the particulars taken into consideration for the optimal formulation of a probe with PFCs or fluorinated molecules are depicted in Figure 2. In short,

1. Significant biological stability and possessing desired chemical traits [91]. The probe must be chemically inert to such an extent that it can endure all of the omnifarious chemicals in the biological milieu until it performs its mission and will be degraded. Most organofluoride compounds easily match this precondition given the strong C–F bond.
2. An ideal tracer should possess a restrained *T*_1_ relaxation time (reduce acquisition times and increase the number of scans per unit time) and an adequately long *T*_2_ relaxation time (to avoid signal intensity loss) [116]. A constant relaxation is anticipated in the complex biological environment. *T*_2_/*T*_1_ ratio close to unity is desirable for a better SNR. One of the drawbacks of PFC is their long *T*_1._ When a PFC has intrinsically long *T*_1_ relaxation (PFOB and PFCE have *T*_1_ relaxations > 1 s), it will severely limit the rate of data acquisition and its sensitivity [98]. Invariably, in the literature, *T*_2_ is an easily manipulable parameter, and this is inspected by modulating the length of fluorinated chains in the probe.
3. High number of magnetically equivalent ^19^F-content: ^19^FNMR spectrum, a characterization technique used in the initial analysis, for an ideal CA should be simple, preferably with a single, sharp, narrow resonance and intense peak to maximize sensitivity and prevent chemical shift imaging artifacts. The integral of an NMR signal is quantitative [117,118], directly proportional to the imaging agent concentration. The probe should also have a high fluorine content to give a single dominant signal and a good sensitivity, customarily accomplished employing PFCs. One of the undesired attributes of PFCs is that some of them lack proper symmetry, so they have a split signal in the NMR due to the disparate chemical environment of the fluorine in the molecule. This issue is surmounted by methodically applying ^19^F MRI probes with high symmetry like PFCE/PFPE or polymeric species like dendrimers.
4. Prominent SNR enhancement: ^19^F MRI often suffers from low SNR [119]. The commonly performed strategies to enhance the SNR are to use a CA, modulate the magnetic field strength [120], to improve pulse sequences [121] or hyperpolarization techniques such as dynamic nuclear polarization, chemically induced dynamic nuclear polarization, spin-exchange optical pumping, and parahydrogen-induced polarization that can achieve the same goal [122]. In the case of ^19^F MRI, using the fluorinated component CA with a considerable amount of fluorine in the probe is also one of the commonly used approaches. Howbeit, high concentrations of CAs might potentially result in toxicity issues.
5. Nominal/no in vitro and in vivo toxicity: neither should it modify any biological functions nor degrade to give by-products detrimental for other tissues/organs and hence should possess low immunogenicity.
6. Easy and scalable synthesis and formulation of CA: a reproducible synthesis that can sustain the purity of the formulation with as simple as a single-step reaction and adeptness of scaling up.
7. Water solubility would be an advantageous feature that would help in the easy application of fluorine. The approach to effectuate water-soluble fluorinated moiety is by chemically modifying the system with hydrophilic compounds or employing hydrophilic hyperfluorinated organofluorine molecules [123]. One requirement for such a probe is possessing a suitable conjugation site. One of the most explored PFC in this regard is PFPE.
8. A long shelf life is favoured for a probe (at least six months).
9. Finally, it is always preferred to have an easy clearance from the living system to be approved for clinical application.

### 4.5. Biomedical Applications of PFC Molecules

PFC emulsions are flourishing in an array of biomedical applications, including molecules with high oxygen solubility for respiration and blood substitution, anaesthetics, chemotherapeutic agents, etc. Time after time it is being used in inflammation studies. Some of the early biomedical applications of PFCs include approved use as artificial oxygen transport vehicles and blood substitutes for human use, as PFC can readily dissolve oxygen and, at a constant temperature, the concentration of O_2_ in the liquid PFC linearly correlates with the partial pressure of O_2_ [124]. Hence, the safety profile of PFCs inside the human body is vastly explored. It is also capable of dissolving carbon dioxide and nitrogen. Over and above, incorporating other imaging agents like fluorescein isothiocyanate (FITC), Alexa647, and boron-dipyrromethene (BODIPy) can further extend their application in the field of multimodal imaging. The unique characteristics of PFCs in unison with their hydrophobic nature favored them to be suitable for US imaging as injectable emulsions of PFCs [125]. Some of the PFC-containing formulations are approved by the FDA for CAs in the ultrasound. They are Definity^®^ and Optison^®^ [126,127], both of which avail PFP/perflutren in the gaseous state [30]. Alike, there are two commercially available PFPE emulsions used for in vivo ^19^F imaging in cell tracking studies–cell sense and V-Sense [128,129,130,131]. Perftoran^®^, rebranded under the name Vidaphor ^TM^, is a drug approved for clinical application in Russia, Mexico, Kazakhstan, Kyrgyzstan, and Ukraine, and is in the progression to be introduced in the US and European markets [132,133], to be used as a blood substitute [134]. It consists of PFD and perfluoromethyl cyclohexyl piperidine as PFCs and is stabilized by proxanol-268-polymeric surfactant and electrolyte mixture [85,133,135].

A plethora of studies are underway to evaluate the various properties of PFCs. They are found feasible for applications such as hypothermic total or partial liquid ventilation of the lungs [135,136,137,138,139], in vivo visualization of the effects of antibiotic therapy [140], oligonucleotide therapeutics [141], cell tracking [142], including stem cells [143,144,145], specific detection of organ rejection [146], identifying penumbra in stroke patient [147], quantifying immune cells (tumour-associated macrophages) in the tumour microenvironment [148,149,150], organ preservation [151], quantifying renal vascular damage [152], ^19^F-oximetry [153], inflammation imaging of various diseases [154,155,156,157,158,159], gasification-enhanced photoacoustic cavitation [160] etc. There are some excellently written reviews for further reference on PFCs used for oxygen delivery [55,63,73,124,161,162,163,164], imaging inflammation [48,91,165,166], and cell tracking [65,98,129,167,168,169]. Refer to the reviews for profound understanding on ^19^F MRI used in biomedicine [44,47,56,170,171,172], PFCs used for various applications [53,74,84,89,173,174,175,176] and fluorinated compounds including PFCs used for imaging and/or drug delivery [17,24,54,177,178,179,180,181,182,183]. Being in the tight grip of SARS-CoV-2, PFCs have been proposed as a source of gas exchange in patients in critical conditions and be employed to protect blood cells [184].

## 5. Examples of Nanosystems Used for ^19^F MRI Studies

A miscellaneous collection of NP probes has evolved and is employed to overcome the present limitations and drawbacks of the ^19^F MRI CA. These nanosystems will be reviewed scrupulously, in particular, nanosystems loaded with PFCs besides fluorinated molecules like dendrimers and polymers will be considered in dept. With the intention to make the study more comprehensible and coherent, the nanosystems have been broadly classified as organic, inorganic, and hybrid systems. The organic nanosystem comprises polymeric, hyperbranched, dendrimer, hydrogel, lipid, and micelle systems. The inorganic system consists of metal and carbon-based nanoparticles. The hybrid or the mixed system consists of a fusion of organic and inorganic systems.

### 5.1. Organic NPs

#### 5.1.1. Polymeric NPs

**Figure 3:**
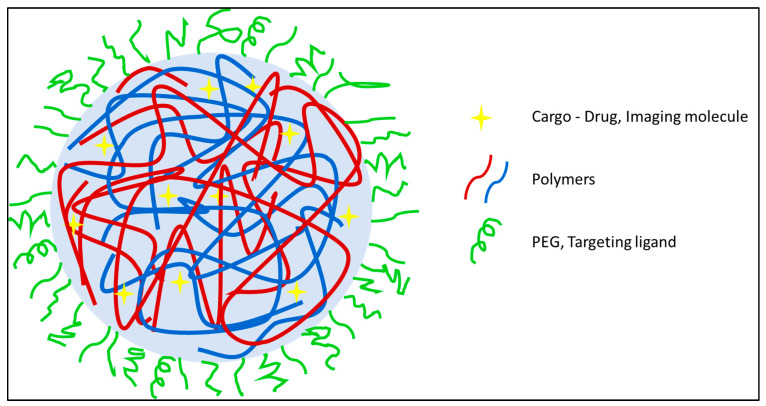
Polymeric nanoparticle complex with random polymer (blue and maroon) coils and a modifiable surface PEGylated surface here (green)). They can encapsulate/hold the payload (yellow star) in the polymer matrix.

Due to the sparsity of ^19^F nuclei per molecule, ^19^F MRI possesses a low sensitivity which subsists as the major stumbling block. These, in turn, can help in increasing the sensitivity, thus reducing chemical shift artifacts. Figure 3 represents a general polymeric nanosystem where the NPs are pieced together with either one polymer or a combination of two or more polymers that can encapsulate the payload with a manipulable outer surface. Such a system increases the practicability to make it chemically fine-tuned, smart NPs that hold the possibility to add drugs moieties or target ligands compliant with its physicochemical properties.

The additional advantages of using polymeric species include high stability, manipulative chemical synthesis based on the desired properties, and depending on the polymers, they can be easily eliminated from the body [185,186]. In polymeric NPs, generally, two types of system are encountered-nanosystems made of CA-modified polymers that are fluorinated polymers and physical encapsulation of CA (mainly using PFCs) into nanosystems.

Geared towards overcoming the quandary faced by typical PFC encapsulated NPs–emulsion localized in diseased tissue and long-term accumulation in off-target tissue, Wallet et al. prepared low-molecular-weight fluorous polymeric colloidal NPs [187]. The NPs from the copolymer synthesized by atom transfer radical polymerization (ATRP) using an azide-terminated initiator consisted of trifluoroethyl methacrylate and oligo (ethylene glycol) methyl ether methacrylate, and they proved effective for breast and ovarian cancer models with little off-target accumulation. NPs have been prepared from ^1^*H*,*H*-perfluoro-*n*-octyl acrylate, *N*-vinylformamide, (1,5-*N*-vinylformamido) ethyl ether, and (*E*)-2,20-(diazene-1,2-diyl)bis(2,4-dimethylpentanenitrile as an initiator by one step, free radical polymerization technique [188]. The NPs appear to be promising carriers for ^19^F MRI CAs, and their in vivo and ex vivo studies are yet to be carried out to confirm the results. A salient amalgamation trio of the fluorophilic−lipophilic−hydrophilic system was developed by Kaberov et al. using poly(2-oxazoline) block copolymers [189] to be an imminent ^19^F MRI CA. Di- and tri-block-low molecular weight copolymers were synthesized based on 2-(^1^*H*,^1^*H*,^2^*H*,^2^*H*-perfluorooctyl)-2-oxazoline, 2-methyl-2-oxazoline, and 2-*n*-octyl-2-oxazoline, which self-assembled in aqueous solution and DMSO to micelles like structure and polymersomes possessing a core–shell structure.

A new class of fluorinated MRI agents, synthesized by one-pot reversible addition-fragmentation chain transfer (RAFT) polymerization of ^19^F-containing functional copolymer poly(oligo(ethylene glycol) methyl ether methacrylate-*co*-2,2,2-trifluoroethyl acrylate-*b*-poly(styrene-*co*-3-vinylbenzaldehyde) (poly(OEGA-*co*-TFEA)-*b*-poly(St-*co*-VBA)) was introduced to study the effect of morphology on different properties of NPs [190]. The core−shell structured particles formed from the copolymers proved that polymeric nano-objects of varied morphologies could be potential ^19^F MRI agents potential ^19^F MRI agents. The prepared NPs were of spherical, worm-like, or vesicle particles morphologies. Comparative studies showed that worm-like NPs had the highest uptake, and vesicle NPs were less likely to be taken up by the cells as the former has a higher aspect ratio. Levels of cytotoxicity (Chinese hamster ovarian cells) were in the order of spherical NPs > vesicle NPs > worm-like NPs, although none of them were toxic, while ^19^F MRI sensitivity was in the order of spheres > worm-like > vesicles (which depends on the fluorinated segment’s motion in the corona). Interestingly, there was not any variation in the *T*_2_. This study showed the influential role of morphologies in NPs.

Fu et al. developed novel ^19^F polymeric imaging agents activated by reactive oxygen species (ROS) such as H_2_O_2_ or low pH [191]. The monomers (thioether- and fluorine-containing methacrylate) were gathered from a PEG-based initiator by ATRP. The NPs formation was ensued in an aqueous solution by self-assembly with compact hydrophobic cores to give core–shell structured nanoaggregates. The imaging agent acted as a molecular switch by variation of *T*_2_ relaxation in the presence of ROS, depending on the oxidation of the hydrophobic thioether group of the agent into hydrophilic sulfoxide group. These were pertinently useful for imaging cancer cells as the environment is hypoxic. For specific imaging of bio-thiols using ^19^F MRI, Huang et al. developed intracellular reducing microenvironment-induced amino-activatable nanoprobe [192]. The copolymers for the nanoprobe acquired by RAFT polymerization from 2-((2,4-dinitro-*N*-(3,3,3-trifluoropropyl)-phenyl)sulfonamido)-ethyl methacrylate monomers. This nanoprobe could specifically detect bio-thiols, including cysteine, homocysteine, and glutathione. Initially, the fluorinated segments immobilized in the hydrophobic core quenched the MRI signal (OFF state). When encountered a sulfhydryl moiety, a change induced in the molecular substitution of the nanoprobe ultimately dissembled the nanoprobe, and MRI signals were regained (ON state).

To evaluate the influence of NP’s charge on the stability of ^19^F MRI CAs, a fluorinated multifunctional monomer was used to prepare cationic NPs. The study started up with the development of six forms of NPs with poly(methyl methacrylate) as the hydrophobic block and differing in hydrophilic block [PEG, mannose, fructose, two different 2,2,2-trifluoroethylamide L-arginine methacrylamide ratios (10 and 20 mol%)]. This study concluded that the choice of the hydrophilic copolymer had an immense implication on stabilizing the NP corona, thereupon the performance of the CA. The conclusion stemmed from the finding that PEG with bulky side chains prevented the aggregation of the fluorinated moieties in the NP corona, hence exhibiting extended *T*_2_. Other studies in the polymeric system include Nafion (sulfonated tetrafluoroethylene based fluoropolymer-copolymer) based nanocarriers that were experimented with for ^19^F MRI [193] together with poly-*L*-lysine and pegylated poly-*L*-glutamic. Cascaded, multi-responsive, self-assembled nanoprobe was identified for sensing and imaging by the sequential redox-triggered and NIR irradiation-induced ^19^F MR signal activation/amplification [66]. The nanoprobe consisted of amphiphilic polymers containing monodisperse PEG (mPEG_2k_) and ^19^F bearing moiety with NIR-absorbing indocyanine green (ICG). The ICG NPs dissociated in a reductive environment leading to the formation of ultrasmall NPs that could further dissociate to small and water-soluble molecules under the response to photothermal therapy.

Srinivas and co-workers have harnessed the benefits of poly(D, L-lactide-*co*-glycolide) (PLGA) (Resomer RG 502H, lactide: glycolide molar ratio 48:52–52:48) particles of sizes ranging from 200 nm to 2000 nm formulated by single and double emulsion techniques under sonication [194]. The effects of the moiety confined inside the NPs—different PFCs, and the surface coating (targeting agent, antibody)—have been studied and shown that the NPs were exceedingly flexible in terms of encapsulated contents (imaging agent, fluorescent dye, drug), particle size, charge (−40 to 30 mV), and the bound moiety. These resulted in a versatile system with the capability to optimize parameters depending on the application. PLGA NPs were already documented for the detection of the labeled cells and direct quantification of cell migration in a diabetes model, using the PFPE by the cellular MRI method with the anticipated expansion of the PFPE-imaging platform to a wide range of cell and disease models. It was also proposed that the PFPE imaging platform could be outstretched to a wide range of cell and disease models [195].

PFCE/perfluron with different fluorescent dyes were encapsulated inside the PLGA NPs for simultaneous imaging of distinct cell populations [196]. The PLGA-PFCE-NPs with ICG or fluorescite fluorescent dye was applied simultaneously to obtain the images in less than ten minutes, making it expeditious. The slow processing and poor resolution images from unsatisfactory penetration of MRI were prevailed over by fluorescence imaging. PFCE encapsulated PLGA NPs were recognized to have many applications such as imaging using ^19^F MRI in conjunction with the US discerning the NPs were stable on exposure to high-pressure ultrasound [197]. With ICG dye incorporated in the formulation, its application was extended to PAI and fluorescent imaging [198] and to obtain cardiac ^19^F MRI using PFCE labeled cells [199]. These PLGA-PFCE-NPs were used alongside gold NPs to assess the bone fillers and images using MRI and CT [200].

Srinivas et al. did an exhaustive study with PLGA (resomer RG 502H, lactide: glycolide molar ratio 50:50) for the formulation of triphasic NPs containing PFCs applicable to clinical imaging [201]. Exploring different parameters such as surfactant type and concentration, polymer concentration, and solvent type affecting the miniemulsion formation of PLGA NPs loaded with PFCE, such as their size, stability, release properties, and cell viability. The insight on the ultrastructure of the NPs is crucial for determining their exclusion from the body. The PLGA-PFCE NPs established to have a multicore structure in contrast to the anticipated simple core one, which helped in the easy clearance of NPs from the body, as proven by in vivo studies conducted on mice [202]. The simultaneous loading of two PFC agents (PFCE and PERFECTA) yielded a two-color MRI probe [203]. When modified with ^111^In-DTPA to the PFCE-PLGA NP, they had the aptness for combined SPECT/PET and ^19^F MRI in vivo cell tracking [204]. Since these particles are powerful theranostic agents evidenced from the previous discussions, their production was aspired to be scaled up. It was achieved by a modular microfluidic system, with sufficient yields for clinical use [205].

Chitosan-coated PLGA-PFOB NPs (RG Resomer 504H) attained by homogenization under emulsion evaporation method followed by sonication was applicative for tracking in vivo cell migration [206]. The encapsulation efficiency of PFOB was 67.1% ± 10 (*w*/*w*). Cyanine dyes like IR Dye 800CW used are advantageous for biomolecule labeling and in vivo clinical diagnostic. NPs derived using single emulsion and solvent extraction methods with NIR fluorophores and PFCE give an entrapment efficiency of PFCE of around 240 μg/mg. The same group prepared PLGA-PEG-folate polymer, encapsulated with PFOB and either ICG (for NIRS) or the chemotherapeutic agent doxorubicin, showed enhanced uptake on human nasopharyngeal epidermal carcinoma (KB) cells, and in vitro cytotoxic studies showed that folate-targeted NPs were able to kill cancer cells more efficiently than non-folate conjugated counterpart [207]. With an encapsulation efficiency of 80% PFOB, the same NPs had been taken advantage of rheumatoid arthritis diagnosis [208]. Poly(styrene sulfonate), an ionic polyelectrolyte polymer, was used to modify the PLGA-PFOB NPs, to be used for cell labeling [209]. It was demonstrated from the in vivo and in vitro studies that the prepared NPs could be effective for cell tracking studies with MRI, least affecting any cellular functions.

PEGylation of PLGA polymer is a widely used approach for increasing the half-life of the NPs in the bloodstream, and it was reestablished with PLGA-PEG nanocapsule encapsulated with PFOB [210]. For probing ultrasound-triggered drug release, PFOB loaded PLGA-PEG NPs encapsulated with Nile red had been investigated, which proved that the mentioned NPs are least suitable for the function due to the requirement of robust inertial cavitation [211]. Cruz et al. manipulated PEGylated PLGA NPs for the detection and monitoring of ischemic diseases and traumatic brain injury, using optical microscopy and ^19^F MRI [212]. Tumour-associated macrophages (TAM) are tumour-promoting inflammations that could be potential biomarkers for diagnosis, prognosis, and therapeutic targets for cancer [213]. Zambito et al. used PLGA-PEG-mannose NPs encapsulated with PFCE to visualize TAMs by optical imaging and ^19^F MRI [214] with higher specificity and robust signal strength. NIR dye encapsulated PLGA-PEG NPs were adapted for monitoring and imaging in osteoarthritis by modifying the NPs with trifluoroacetamide [215]. In vitro, in vivo and, ex vivo ^19^F MRI and optical imaging studies proved their prospect to be multi-modal nanoprobes.

The combined effect of PFCs capability to diffuse oxygen into the tumour tissue and the possibility to modify the surface of PLGA NPs have been exploited for the enhanced antitumour efficacy in colon cancer using PLGA NPs functionalized with epidermal growth factor and co-loaded with 5-fluorouracil (chemotherapeutic drug) and PFC [216]. The aforementioned system proved to be more fitting in accumulating in tumours via ligand-targeting interactions and amended the hurdle of hypoxia-induced chemotherapy resistance. A very distinct approach was adopted by Neri et al. for the PLGA polymers-they had been fluorinated with two different fluorinated amine ligands (coupling reaction) to form F_3_-PLGA and F_9_-PLGA that contained three and nine equivalent fluorine atoms, respectively [217]. They displayed a higher efficacy to load hydrophobic drugs. Preliminary in vitro studies of F_9_-PLGA NPs were done using the drug (dexamethasone) loaded NPs to assess their cellular availability and drug release showed a greater efficacy.

The linear PFPEs possess a functional group, unlike most PFCs, to allow for facile chemical modification, and this trait was maneuvered to achieve the desired effect. A nanoemulsion with tyramide modified PFCE with NIR dye, surfactants, and hydrocarbon oil was designed for hydrophobic drugs delivery and dual imaging [88]. By inhibiting the function of the cyclooxgenase-2 enzyme by selective inhibitor-Celecoxib is an anticancer strategy to reduce cancer risk and suppress tumour growth. Janjic et al. have reported the PFPE nanoemulsions loaded with Celecoxib and NIR dye for theranostic application including, three complementary imaging modalities-fluorescence, NIRS, and ^19^F MRI [218,219]. The application of the developed nanoemulsions has been extended for in vivo monitoring and modulating tumour-infiltrating immune cells [218]. The authors were the first to show the two-color PFC nanoemulsion [220]. PFPE was modified with oligo(ethylene glycol) methyl ether acrylate by RAFT polymerization to form a CA that had high imaging sensitivity and was hydrophilic [221]. To investigate the aggregation behavior of nanosystems that can have a role in the interaction between the NPs and living entities, doxorubicin-loaded polymeric PFPE-based NPs were reported [222]. Evaluation of fluorinated NPs on 3D spheroids concluded that for greater efficacy of drug delivery, it was efficacious for the NPs to have a smaller fluorinated core and the fluorinated segments to have greater exposure to the external environment.

RAFT polymerization intended to combine 2,2,2-trifluoroethyl acrylate with 2-(methylsulfinyl)ethylacrylate resulted in an MRI CA that was exceedingly hydrophilic and displayed intense in vitro/in vivo MRI signals [223]. With the solid-phase peptide synthesis of disordered fluorinated peptides by sequential addition of amino acid–trifluoroacetylated lysine, a platform conceivable for in vivo targeting applications was made [224]. Copolymers developed from perfluoropolyether methacrylate and oligo(ethylene glycol)methacrylate and modified with a green fluorescence dye–*N*-(5-fluoresceinyl)maleimide, had a hydrodynamic size around 12 nm and molecular weight ~75,000 gmol^−1^ [225]. From the in vivo studies, it was acknowledged to have favorable non-phagocytic cells uptake profiles and outstanding MRI performance. With the widely used PEG, a novel, low cost, hydro-soluble, highly flexible, easily tunable with a facile synthetic route, PEG-based fluorinated esters were built up using 2-(trifluoromethyl)-3,3,3-trifluoro-propanoic acid [226] and PFTB [227]. Polydispersity in PEG is an inherent trait of the polymer, and recent years have seen the development of mPEG with improved biodegradability [228]. A thermoresponsive imaging probe with fine-tunable lower critical solution temperature pioneered from peptidic mPEG combs [229] was explored for their smart drug-carrying ability using doxorubicin.

#### 5.1.2. Hyperbranched

**Figure 4:**
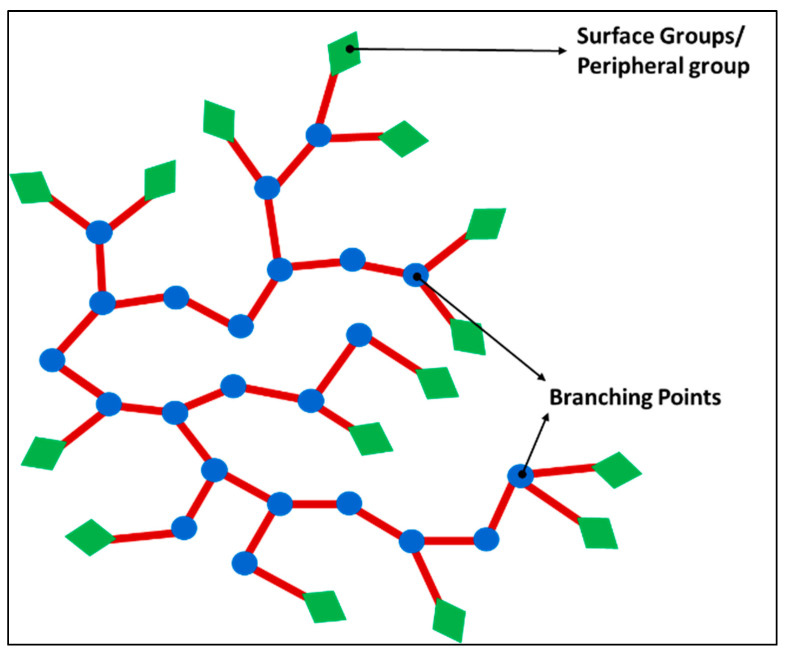
Hyperbranched polymers structure - the polymer structure is a randomly branched polymer with circles (blue) representing the branching points and rhombus shapes(green) for surface groups.

Coupled with linear, cross-linked, and branched-chain polymers, dendritic polymers are the fourth subclass of polymers that are invariably branched irregularly [230]. Hyperbranched polymers (as shown in Figure 4) are a subclass of dendritic polymers whose polymeric structures are bestowed with abundant functional groups, intramolecular cavities, low viscosity, and high solubility [231]. This class of molecules has been ventured mainly to overcome the intrinsic drawback of PFC formulations like low stability, limited aqueous dispersibility, and a limited possibility to functionalize.

As already highlighted regarding the benefits of multimodal imaging–combining the high resolution, 3D anatomic images for soft tissues with MRI, and high spatial resolution for hard tissue by CT, a more accurate diagnosis is guaranteed, facilitating treatments. Multifunctional hyperbranched polymers containing iodine and fluorine were synthesized by initially using a hyperbranched iodopolymer via RAFT polymerization. The 2-(2′,3′,5′-triiodobenzoyl)ethyl methacrylate was incorporated to provide X-ray opacity along with poly-(ethylene glycol) methyl ether methacrylate (PEGMA) to provide hydrophilicity, and bis2-(methacryloyl)oxyethyl disulfide was chosen as a crosslinker to achieve biodegradability [232]. HBIP was chain extended with 2,2,2-trifluoroethyl acrylate (TFEA) and PEGMA to obtain hyperbranched iodopolymer containing ^19^F (HBIPF). From the in vivo degradation studies, the polymers were proven to be biodegradable. Thereby, this study demonstrated that multifunctional hyperbranched polymers were promising molecular imaging agents for CT/^19^F MRI bimodal imaging.

To boost the local fluorine concentration, segmental mobility of the fluorine-containing moieties, and for active and specific targeting of diseased tissues, a multifunctional PFPE-based NPs conjugated with a peptide aptamer, Hsp70, as targeting ligand was pioneered (Hsp70–specifically to target the heat shock protein 70 overexpressed in breast cancer cells) [233]. They were attained from RAFT polymerization with hydrophobic PFPE segments and oligo(ethylene glycol) methyl ether acrylate (OEGA) as the hydrophilic monomer. The poly(OEGA)_3_-PFPE polymer, further chain extended with OEGA and ethylene glycol dimethylacrylate (EGDMA) monomers gave rise to a hyperbranched PFPE-based polymer. After polymerization, fluorescence dye molecules, Cy5.5 were conjugated by reduction at the termini and aptamer peptide by click chemistry. The series of outcomes from the in vivo detection of breast cancer on a murine tumour model indicated that PFPE based NPs are efficacious theranostic agents for the specific detection of in vivo breast cancer. The explored properties included scrutinizing the ^19^F NMR and MRI properties, in vivo and ex vivo molecular imaging, in vitro cell uptake, intracellular distribution, and trafficking, elimination of polymers from the body alongside tumour-penetration analysis.

Self-assembled colloids prepared using fluorinated hyperbranched polyglycerols were macromolecules germane to therapeutic functions [234]. By ring-opening multibranching polymerization (ROMBP) of glycidol followed by copolymerization with a fluorinated glycidyl ether (2-[(2,2,2-trifluoroethoxy)methyl]oxirane), hyperbranched polyglycerols were formed and explored for their ability to perform both as ^19^F-MRI nanoprobes and drug-loaded nanocarrier. A synthetic steroidal anti-inflammatory drug–dexamethasone, was used as the model drug. The formation of the micelles gave a narrow size distribution after the drugs were incorporated inside.

#### 5.1.3. Dendrimers

**Figure 5:**
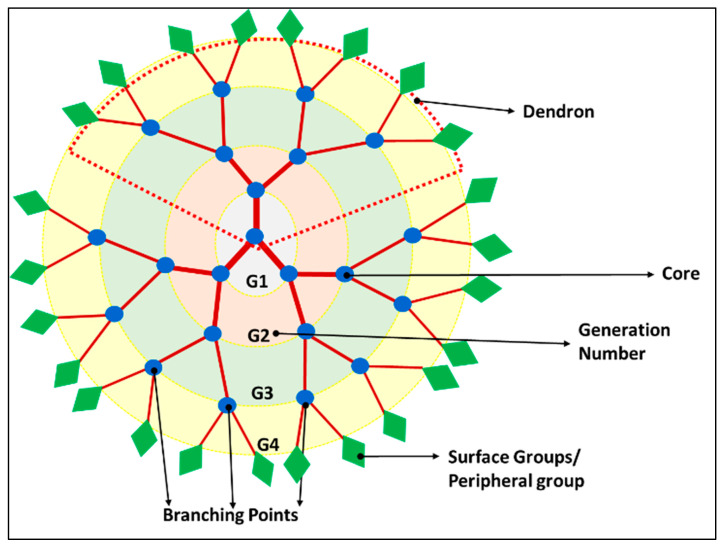
Dendrimers structural components include the core, branching points (blue circles), surface/peripheral groups (green rhombus), and the a dendron segment in a dotted red triangle.

Even though multiple fluorines could be incorporated into a single molecule in fluorinated polymer, they suffer from pitfalls that often split signals are obtained in the FNMR. A group of macromolecules belonging to the family of dendritic polymer is of great use in enhancing the ^19^F signal intensity per imaging agent molecule since they possess a spherical symmetry that can provide an identical chemical environment to the multiple fluorine atoms [235]. Frequently obtained from convergent synthesis methods, they self-organize to form well-defined 3D structures called dendrimers, with radically distributed branches, growing out from a focal point as illustrated in Figure 5. As they flare out to wide branches, the ‘generation’, as well as the number of peripheral groups of a dendrimer can be recognized from each subsequent branching unit. Even though both hyperbranched polymer and dendritic polymer have a 3-dimensional (3-D) macromolecular structure, the difference between them is that the latter has a regular topology as pictorially represented in Figure 5 with a multistep synthesis, while the former has an irregular topology as shown in Figure 4 with relatively facile one-step preparation [230].

Dendrimers have unique properties including monodispersity, multi-valence, uniform and well-controlled size and shape, modifiable peripheral surface groups, and available internal cavities that make them a strong candidate for both imaging and drug delivery [236]. Their internal cavity can incorporate other imaging agents or drugs and have high intrinsic payload capability. When these polymer chains are fluorinated, they are adequate for ^19^F MRI. The first fluorinated dendrimer studied for MRI was a small Janus dendrimer, a polymer assembly with a core attached to two different side chains [237]. Multiple studies had been carried out to study the dendrimers as nanocarriers.

The intricacy of ^19^F MRI is their high *T*_1_, nuclear anisotropy, and frequently, NPs made by emulsions result in a size greater than 200 nm that can hardly pass through the capillaries of the blood vessel [238]. To evade these demerits a bifunctional Gd^3+^ chelate (DOTA—1,4,7,10-tetraazacyclododecane-1,4,7,10-tetraacetic acid) was prepared and characterized to be employed as dendrimers. The dendrons, synthesized using fluorinated amino acids (BOC-L-4-trifluoromethylphenylalanine and 3,5-bis(trifluoromethyl)-DL-phenylalanine) along with carboxylic acids of the repeat branch unit. Different dendrimers with a size around 3 nm and *T*_1_ decreasing with increasing dendrimer generation, were tested with animal studies (Sprague Dawley female rats), and they exhibited less toxicity (KB cells) and had better SNR [239]. Kolmël et al. described the synthesis of polyfluorinated second-generation dendrons consisting of 72 magnetically equivalent fluorine atoms and displaying a single sharp resonance in its ^19^F NMR spectrum. The polymer was prepared by repeating iteratively Sonogashira coupling, alkyne deprotection, and copper-catalyzed azide-alkyne cycloaddition (CuAAC) for the generation build-up [240]. For a plenitude of pseudo symmetrical fluorines and excellent ^19^F MRI properties, the target dendrimer was convergently synthesized on a gram scale over 11 steps with an overall yield of 8%. Through assembling of the building block, the acidic bis(trifluoromethyl)carbinols, 540 fluorines were symmetrically distributed on each spherical layer, in unison emitted a single ^19^F peak with high signal intensity and therefore had high ^19^F MRI sensitivity [241].

Fluorinated self-assembled dendrimers were observed as promising ^19^F NMR/MRI-traceable drug-delivery vehicles for in vivo tracing and quantifying drugs, detecting drug microenvironments, and weak interactions [242]. It was established that co-self-assembly of fluorinated amphiphile dendrimers could determine weak interactions between the drug and the drug-delivery vehicle because of the changes in the self-assembling profile (π–π stacking, hydrophobic interactions, etc.) that sensitively effectuated corresponding ^19^FMR responses. To study drug-amphiphile interactions in micelle- and liposome-based drug-delivery systems, a total of 15 model molecules with structural diversity such as (R)-carvone, cholesterol, the anesthetic propofol, and the anticancer drug doxorubicin, were chosen. In comparison to the per-hydrogenated dendrimers, fluorinated counterparts had different traits due to the fluorophobic effect relative to solubility and micro-segregation effect. Like PFCs, their degradation pathway and toxicities were still ambiguous after being retained for a longer time in the body [242].

Although dendrimers seem like a scintillating prospect, the enigma faced by this class of molecules is their arduous synthetic procedure and use of organic solvents that limit them from being an easily approachable technique. Often, for the formation of dendrimers, different chemical groups can be fine-tuned depending on the outcome. Its cytocompatibility, biodegradability, cellular toxicity, and cellular uptake are complex and abstruse, and on top, it requires further investigations and inferences. There is also a condition called “hydrophobic aggregation-induced signal attenuation” that happens when the ^19^F-content in the molecular structure is greater than 10 *wt*.% [243]. The nanoprobes cannot exceed a threshold concentration of fluorine, for stimulation in biological systems, as exceeding a base concentration result in hydrophobic aggregation of fluorinated segments.

#### 5.1.4. Nanohydrogel

**Figure 6:**
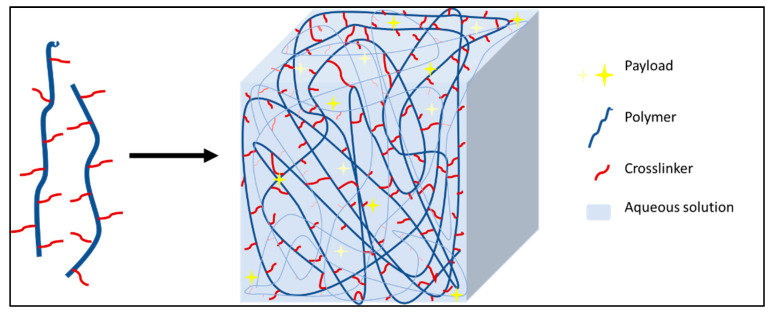
Nanohydrogel matrices are formed by polymers that can form the 3D network with the help of crosslinkers (red) that act as linking ligands. The payloads (yellow star) can be trapped inside the 3D matrix.

Hydrogels are 3D hydrophilic cross-linked or self-assembled polymer networks (Figure 6) that have high loading capacities of payloads (30% wt.), self-healing ability, viscoelastic behavior, ample stability, and can be triggered to release the payload through swelling in response to environmental changes in pH, ionic strength, or temperature [244,245]. The payloads can be encapsulated in nanohydrogels through various means, such as (i) passive/diffusion-based, (ii) covalent conjugation to either the interior or exterior, (iii) physical entrapment within the polymer network [246].

Designing NPs targeting the lymphatic system (a vital part of the immune and circulatory system), both PEGylated and fluorinated chitosan were synthesized to fathom their application for encapsulation of probes and MRI lymphography (relating to the body’s lymphatic system) experiments [247]. The nanohydrogels were prepared by ionic gelation, the spontaneous supramolecular assembly of cationic chitosan with anionic compounds. After resolving the dilemma of determining the degree of substitution of PEGylated and fluorinated derivatives with chitosan, in vivo experiments affirmed good biocompatibility and prospective use of nanohydrogel for the relevant applications. Similarly, a thermoresponsive hydrogel was reported by Kolouchova et al. where the structure of the nanohydrogel was based on amphiphilic copolymers containing two blocks: one hydrophilic biocompatible block–poly[*N*-(2-hydroxypropyl)methacrylamide] (PHPMA) or poly(2-methyl-2-oxazoline) (PMeOx) and one fluorinated thermoresponsive block–poly[*N*(2,2difluoroethyl)acrylamide] with excellent sensitivity and non-cytotoxic for cell lines like human cervical carcinoma, murine monocyte/macrophage, HF-primary fibroblasts, and human B lymphoblast cell lines [248].

To prevail over the crucial challenge of aggregation in fluorocarbon substitutions that induced the segments of polymers hydrophobic, Munkhbat et al. had used an intelligent chemical play using nanohydrogels [249]. It facilitated in fully realizing the potential of polymeric tracers. Firstly, polymeric assembly was constructed with degradable hydrocarbon moieties and a high fluorocarbon core, and by chemical cross-linking, preserved the morphology of assembly. Eventually, segmental mobilities were amplified within the nanohydrogel interior by triggered degradation of cleavable hydrocarbon parts that decreased the density of the assembly’s interior. That prompted escalated *T*_2_ relaxation time and propelled signal intensities enhancement in ^19^F NMR and ^19^F MRI phantom imaging.

To delve into the controlled release of bioactive agents, ^19^F MRI was used to quantify the degradation rate of implantable or injectable hydrogels and provide the precise location in a real-time and non-invasive manner, without interruption of endogenous background signals and limitation of penetration depth. Traditionally, gravimetric methods are being used to provide this information in vitro but offer limited insight on the in vivo fate and sequential tracking [250]. Ergo, a zwitterionic, fluorinated and alkynyl ^19^F MRI molecular CA was designed, namely *N*-(carboxymethyl)-*N*-methyl-*N*-(3,3,3-trifluoropropyl) prop-2-yn-1-aminium (termed PA-CBF_3_), with zwitterionic carboxybetaine structure, which was superhydrophilic and had superior resistance to protein adsorption and was capable to tether with different hydrogels [243]. The probed nanohydrogels included polyacrylamide hydrogel, injectable alginate hydrogel, thermosensitive poloxamer hydrogel, and poly(ethylene glycol)-*b*-poly(L-valine) polypeptide hydrogel.

Besides manoeuvring of PFPE modified polymeric nanoemulsion for various applications [65,88,94,163,218,219,220,251], Janjic et al. had extended their use in hydrogels too. Anti-tumour necrosis factor-alpha (anti-TNFα) therapy had been a proven strategy for treating inflammatory bowel disease, where TNFα-binding lactococci bacterium can also act as infrared fluorescent protein. For localized delivery of anti-TNFα therapy, the PFPE nanoemulsion loaded with theranostic TNF α-binding lactococci (*Lactococcus lactis*) was incorporated into a thermoresponsive polymer (Pluorinic^®^F127) hydrogel [77]. The resulting nanoemulsion-based hydrogel (nanoemulgel) was ^19^F MRI and NIRS visible. The same group had used a slightly modified hydrogel for increasing its ability to load different payloads (fluorescent dyes, pH sensors, chelators, drugs, and antibodies) and therefore adapt the hydrogel for a broad range of biomedical imaging and delivery applications. PFPE nanoemulsions were crosslinked with polyethylenimine to form hydrogels hence ridding the necessity of any energy utilizing the emulsification step [252].

#### 5.1.5. Lipids

**Figure 7:**
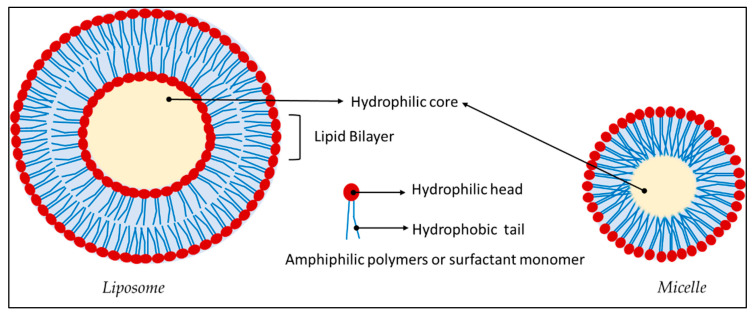
Liposome and micelle consist of an assembly of amphiphilic polymers or surfactant monomers that possess a hydrophobic tail (blue) and a hydrophilic head (maroon).

The primary component for the vaccine technologies (Pfizer/BioNTech and Moderna) used during the outbreak of the novel coronavirus causing severe acute respiratory syndrome is lipid NPs [253]. This system helped in the translocation of the mRNA/self-replicating RNA (responsible for producing the immune response) across the plasma membrane. Typically, liposomes are derived by the self-assembly of the phospholipids like phosphatidylethanolamine, phosphatidylcholine, phosphatidylglycerol, or cholesterol. As portrayed in Figure 7*,* they possess a hydrophilic core and a lipid bilayer, thereupon have the stupendous advantage over other NPs in encapsulating hydrophilic (in the core), hydrophobic (between the bilayer), and even amphiphilic drugs in addition to the possibility of surface modification [175]. They are one of the widely used systems with less known toxicity compared to conventional drugs. For instance, they are the primary component of the first FDA-approved nanodrug Doxil^®^ for the treatment of Kaposi’s sarcoma, ovarian, and breast cancer [254].

Cellular therapeutic dendritic cells (DC)-based vaccination is an ex vivo modified DC with tumour-associated antigens and had been used to initiate anti-tumour immune responses. Hence, it is vitally substantial to track the fate and location of the injected DC. Intending to collectively load antigenic proteins into DC and enable high-resolution tracking of the antigen-loaded cells ^19^F-MRI CAs, antigen-coated PFC particles for DCs were prepped. The cationic particle consisted of 1,2-dioleoyl-3-trimethylammonium-propane, 1,2-dioleoyl-sn-glycero-3-phosphoethanolamine, 1,2-distearoyl-sn-glycero-3-phosphoethanolamine-N-[amino(polyethylene glycol)-2000], and cholesterol. The PFC component was either PFH or PFCE, and it was emulsified in the presence of excess lipid solution through high frequency shaking. Particles were loaded electrostatically by negatively charged ovalbumin (commonly used model antigen) [255]. The same particles were used for in vivo imaging the transplantation of pancreatic islets and tracking the autoreactive T-cell migration in the pancreatic region [256]. Liposomes were formulated with hydrophilic organofluorine molecules with a fluorine encapsulation up to 22.7 mg/mL [257] that could concomitantly image multiple targets without any chemical shift artifacts.

In an attempt to mimic the temperature range in tumours (37–39 °C) that has a different microenvironment than normal cells, Lima et al. demonstrated the change of the ^19^F NMR signal of F—containing compound in thermally responsive lipid nano-emulsion particles, mainly the *T*_1_ and *T*_2_ values, depending on the temperature change (37–42 °C). The carriers were tripalmitin, tristearin, and triarachidin, favored based on high melting point neutral saturated fatty acid, and the fluorine compound was a modified α-tocopherol. The study concluded that *T*_2_ changed more than *T*_1_, and the change in *T*_2_ was mainly given by the increased molecular motion of the modified α-tocopherol, highlighting that the local temperature might impact the ^19^F NMR signal intensity [258]. With MRI multimodal imaging, it was demonstrated that PFC NPs were prospective to be delivered for lung cancer [259]. The PFCE emulsions with rhodamine-phospholipid surfactants consisted of 20% (*v*/*v*) PFCE, 2% (*w*/*v*) of a surfactant commixture, 1.7% (*w*/*v*) glycerin and water. Surfactant commixture consisted of dipalmitoylphosphatidylcholine, cholesterol, Gd-diethylenetriaminepentaacetic acid-phosphatidylethanolamine, 1,2-dipalmitoyl-sn-glycero-3-phosphoethanolamine-*N*-(lissaminerhodamine *B* sulfonyl), and 1,2-dipalmitoyl-sn-glycero-3-phospho-(1’-rac-glycerol). The PFCE emulsions were exposed to human bronchial epithelial (BEAS-2B) and human lung squamous carcinoma (H520) cell lines following intratracheal or intravenous administration. This study established evidence that with minimal extratumour systemic exposure, PFC NPs can be locally delivered into lung cancers intratracheally (reported for the first time) in high concentrations.

#### 5.1.6. Micelle

Typically, micelles are obtained via the self-assembly of the amphiphilic molecules similar to liposomes. They engineer into the core–shell architectures that possess outer hydrophilic surfaces that can impart steric stability, and prolong their circulation lifetimes while the entire interior of the NP is hydrophobic, which portends that only hydrophobic drugs could be encapsulated as depicted in Figure 7 [175]. This scenario leaves hydrophilic cargo to be attached to the surface. They are smaller (10–100 nm) than liposomes, making them suitable for leaky vasculature of tumours. A biosynthesized fluorinated protein was presented by Hill et al. [260] as a “fluorinated thermoresponsive assembled protein” (F-TRAP) that could encapsulate small-molecule chemotherapeutic doxorubicin in its self-assembled micelle structure and release them in response to temperature and concentration, owing to its inherent stimuli-responsive properties. They bore a coiled-coil pentamer corona and a hydrophobic, thermoresponsive elastin-like polypeptide core. When exposed to increased concentration and temperature, they assembled into nanoscale micelles characterized by nearly a constant ^19^F *T*_1_ relaxation times and a remarkable decrease in ^19^F *T*_2_ relaxation. Furthermore, through thermally induced in vitro coacervation of the proteins at 45 °C, free doxorubicin was collected in the supernatant. The therapeutic efficacy of the precedent had been assessed in mammalian tumour cells, MCF-7 human breast adenocarcinoma cells, and discerned that it was significantly effective at reducing cell viability.

### 5.2. Inorganic NPs

**Figure 8:**
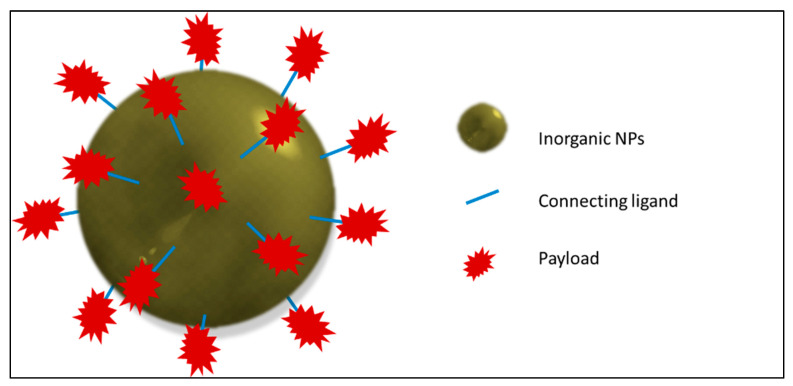
General representation of inorganic NPs where payloads (red star) are attached to the surface of the nanosystem. They include gold, silica, iron oxide, quantum dots, nanotubes etc.

Inorganic NPs such as metallic, magnetic, quantum dots consist of a central core made of inorganic material, as illustrated in Figure 8, which defines their unique characteristics. We would be focusing on metal, silica, and carbon-based NPs in particular, due to the vast literature published for exploring them. Hequet and coworkers [35] had prepared a CA containing a paramagnetic center and chemically equivalent fluorine atoms using a cycloaddition reaction for dual ^1^H/^19^F MRI. *T*_1_ agents (mostly lanthanide complexes) are called positive agents because of the hyperintense signal they engender in the accumulation areas, whereas *T*_2_ agents (usually iron oxide NPs) are “negative” CAs since they induce darker contrast in the accumulation area. A series of cyclen derivative lanthanide (Gd(III), dysprosium (III), terbium (III), and europium (III)) complexes associated with nine chemically and magnetically equivalent fluorine atoms were synthesized, and the study showed that gadolinium, dysprosium, and terbium complexes were promising transition metals for future use in ^19^F MRI in terms of their relaxation time.

Lanthanide-based upconversion (UC) NPs have potential applications in MRI or drug targeting or carriers due to their luminescent properties, such as large anti-stokes shifts, photostability, narrow emission peaks, and low toxicity. NaYF_4_ is an efficient well-known UC host material where Y^3+^ can be replaced in any ratio by rare-earth ions, of which Gd^3+^ is the most attractive one for their intrinsic magnetic properties. To track down the possibility of their UC application in the biological field, water-soluble NaGdF_4_:Yb^3+^/Tm^3+^ nanorods were prepared using the hydrothermal method [261]. The samples were conductive to visible light, and luminescence images were obtained in laser diode excitation. PLGA was reported to encapsulate doxorubicin and inorganic nanocrystals–NaYF_4_:Yb,Er@NaGdF_4_ used for cancer cell imaging and exhibited pH-responsive drug-releasing behavior [262].

Another inorganic material of interest is nanofluoride (calcium fluoride (CaF_2_))-based inorganic nanocrystals. The unique characteristics like controllable content, sizes, and shapes, are often outperformed with the disadvantage of the restricted mobility of the elements within the crystal that leads to NMR line broadening and impedes their use as MRI tracers. Ashur et al. established a synthetic, water-soluble, small (<10 nm) fluoride-based nanocrystals that average out homonuclear dipolar interactions and thus allow high-resolution ^19^F NMR spectroscopy of the nanocrystals in aqueous solutions. The formulated PEG-coated CaF_2_ nanocrystals were used as an imaging tracer, combining the advantages of nanocrystals (small, high ^19^F equivalency, surface modifiability, maximal ^19^F density) with the merits of ^19^F MRI tracer after being used for in vivo ^19^F MRI in mouse models [263]. To increase the relaxivity of NPs that could help improve the SNR, fluoride doped iron oxide (ɣ-Fe_2_O_3_) NPs were considered [264]. Doping citric acid- on the mentioned NPs chemically induced intensification of magnetic anisotropy, unaffecting either its crystal structure or electronic configuration.

#### 5.2.1. Metal NPs

Metal NPs are picked apart for their unique electrical, optical, and mechanical properties [265]. Widely explored for their size, shape, surface chemistry, and optical properties, this class of NPs had opened a broad array of applications when stumbled upon the possibility of obtaining advanced materials with the required properties by various modifications.

The combination of metal NPs with fluorinated ligands anchored on its surface is suitable for ^19^F MRI as this combination of NPs has superior properties in terms of their function. Boccalon et al. [266] prepared gold NPs (F-MPC) for dual-mode imaging, whose surfaces were grafted with fluorinated organic monolayer and ligands ending with a fluorescent dye. The exclusive properties included a central fluorinated chain to generate the ^19^F signal, the ability to solubilize/disperse in water (hence in biological media) without any additives and impart solubility in many solvents due to the presence of terminal hydrophilic triethylene oxide or PEG_550_ chain. Further, the outer monolayer capable to solubilize small hydrophobic molecules laid the groundwork for the development of drug nanovectors. Thanks to these features of the gold NPs that made them a novel imaging platform. The gold core had less than 2 nm size, and the overall size with attached ligands less than 10 nm, permitted them to penetrate smaller blood vessels. F-MPC-cell interactions were evaluated with human cervical carcinoma cells (HeLa), showed that more than 95% of the cells were viable after the uptake.

The aforesaid group had also functionalized the gold NPs with Gd(III) chelates [267] in an endeavor to develop a probe for dual imaging. Some of the impediments considered to get around were the NPs’ detectability and the usual size range of emulsions (200–300 nm), which limit NPs in vivo applications. Increasing the number of fluorine reinforces the odds of making it hydrophobic, hence the development of probes containing enough fluorine to reach detectability in ^19^F MRI is one of the captious challenges in the field. The Gd chelating unit of the ligand was based on the DOTA scaffold, deeply embedded in the monolayer of water-soluble gold NPs that gave good quality MRI images at a 20 mg/mL concentration with Gd(III) units. In pursuance of the ligands with fluorine atoms in the same chemical environment, PFTB ligands were set up for the preparation of gold NPs. Out of the prepared NPs (fluorinated/non-fluorinated, attaching PEG/thiolate to its side chain, etc.), long-chain PEGylated compounds were proven to be the best option to obtain colloidally stable NPs in vitro and hence obtain a single chemical shift, narrow ^19^F-NMR signal, and high fluorine loading [268].

#### 5.2.2. Silica NPs

**Figure 9:**
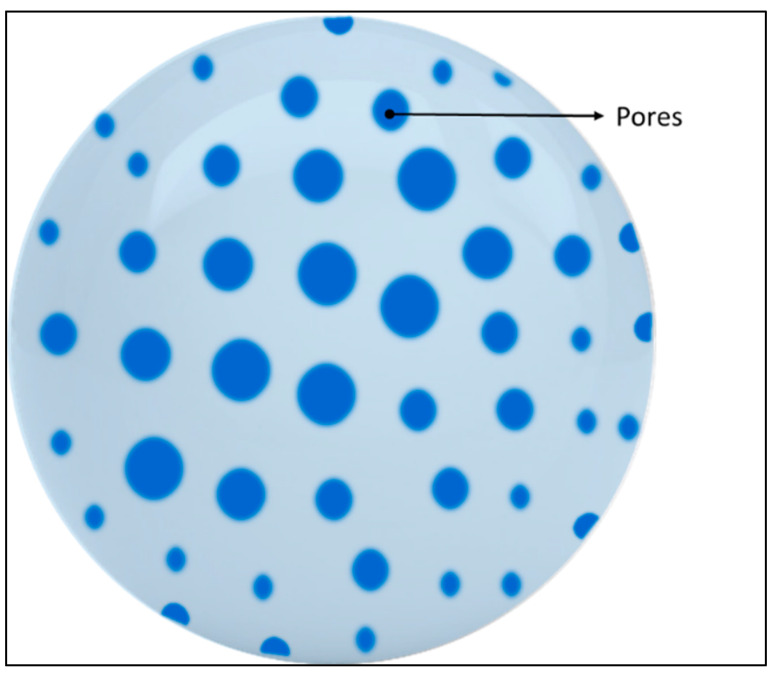
Mesoporous silica nanoparticles structure represented by a blue sphere and the darker small circles shows the pores present on its surface.

Another widely used metal for NPs preparation is mesoporous silica. Massive surface areas (1000 m^2^/g), tunable pore sizes (2–20 nm), facile surface modification capability via various synthetic approaches, and controlled release of numerous drugs from its pore make mesoporous silica NPs (MSN) unique for diversified applications [269]. Hollow mesoporous silica particles filled with PFCE were put to use to demonstrate the type of cargo in the mesoporous silica drug vectors may have substantial influences on the biodistribution [270]. Since the outer surface of these particles is mesoporous as illustrated in Figure 9, the PFCE will be exposed at every stage to the biological environment. Subsequentially, the fate of the NPs can be accurately deciphered from the protein adsorption behavior and how cargo is affecting the protein adsorption. Protein adsorption studies and in vivo ^19^F quantification results proved that adsorbed amount of protein (apolipoprotein A-1 and A-2) was much higher for the PFCE-filled NPs as compared to the native particles and that PFCE-loaded particles were eliminated by liver 72 h post-injection.

Lee et al. have communicated the PFC-loaded ultraporous mesostructured silica NPs (PERFUMNs) for ^19^F MRI detectable oxygen-sensing probes [271]. They had used a post-synthetic loading method for experimenting with three different PFCs (PFCE, PFD, and perfluoro(tert-butylcyclohexane)) which made it possible to encapsulate more PFCs, around five times more than usually encapsulated by MSN. Post-synthetic loading methods were explored to find their influence on the loading yield and efficiency, and it was pinpointed that sonication time is a crucial factor [272]. It was revealed that for silica NPs, as the leaching of PFCs was typical due to their porous structure, it was better to graft fluorine probes onto its surface by covalent bonds. To put this into practice, Bouchoucha et al. [273] reported the synthesis of mesoporous silica NPs with covalent modification with either fluorosilane or polyfluorosiloxane together with a paramagnetic Gd chelate grafted at the surface. NPs (MCM-48-type) were synthesized and functionalized with fluorine-containing molecules and Gd chelates (Gd-DTPA). It helped in the production of imaging probes which induced a strong “positive” contrast enhancement effect, and NPs with the potential for dual ^1^H and ^19^F MRI. Even though the metal Gd on NPs surface aided improvement of the ^19^F relaxation time, a strong effect on *T*_2_ relaxation had the possibility to prevent the detection of probes. This dual detection represented a potential alternative to MRI−PET or in MRI−SPECT and hence the use of radioactive molecules in them.

Kikuchi et al. [274] developed novel multifunctional core–shell NPs utilizing the PRE effect to detect enzymatic (Caspase-3) activity and to use ^19^F MRI for detecting gene expression. Paramagnetic relaxation enhancement (PRE) is the liaison between two magnetic moments of paramagnetic nuclei and observed nuclei, resulting in efficient curtailment of *T*_1_ and *T*_2_ of the nuclei under observation [275,276]. A probe was designed by connecting a ^19^F containing moiety with a Gd^3+^ complex via an enzyme cleavable linker. Anticipating the *T*_2_ of ^19^F would be shrunk by the existing PRE effect in the enzyme reaction (hence low/no signal) as ^19^F containing moiety was close to the Gd^3+^ complex, and when the enzyme cleaved the substrate, ^19^F MRI signal would emanate as *T*_2_ increased in the guise of no longer effective PRE (distance between ^19^F and Gd^3+^ became infinite). However, in mouse experiments with small molecule-based ^19^F MRI probes, there were sensitivity issues, and to prevail over, PFCE inclusive silica-NPs were synthesized with surface modified with PEG.

Being mindful of the ingrained downside of predominant ^19^F MRI probes—arduous modifiability of the surface of nanoemulsions and low sensitivity of small molecule-based probes—multifunctional core–shell silica NPs were introduced for successful detection of gene expression in living cells and tumour tissue in living mice by ^19^F MRI. The biological inertness, favorable colloidal properties, and ease of surface modification of silica with the acquiescence of PFCE resulted in the formation of fluorine accumulated silica NP for MRI contrast enhancement (FLAME) [277]. The surfactant was n-cetyltrimethylammonium bromide (CTAB), and the surface was tailored with a folate receptor. The identic system had been modified for ^19^F MRI traceable silica NPs as drug carriers (mFLAME) by improving the silica coverage of the PFC core, reforming the mesoporous silica shell with a NIR dye (Cy5), and functionalizing it with a folate receptor. By this, it was possible to extend its application to dual-modal imaging (NIRS/^19^F MRI) and drug delivery [278]. Flow cytometric analysis confirmed mFLAME internalized by KB cells (HeLa or cervical adenocarcinoma). Further loading it with doxorubicin had a top-tier cytotoxic effect on KB cells. The combination of fluorine atoms with a paramagnetic ion reduced the ^19^F relaxation times, which was attributed to the PRE effect.

In like manner, to create an OFF/ON switching ability of Ln^3+^ complexes by PRE effect, FLAME NPs were attached to Gd^3+^ diethylenetriamine pentaacetate (DTPA) complexes on its surface by disulfide linkers (FLAME-SS-Gd^3+^). The study had been engaged on the FLAME-DTPA complex after treating with a reducing agent–tris(2-carboxyethyl)-phosphine. This inspection facilitated to show for the first time that the PRE effect of surface Gd^3+^ complexes was effective for fluorine compounds in NPs over 50 Å [279] that were contradictory to the study of De Vries et al., who previously observed that the distance between Gd^3+^ complexes and the fluorine core was less than 22 Å for the PRE of PFCE in Gd^3+^ modified nanoemulsions [280]. FLAME NPs were also reported for their use in multicolor MRI probes (PFC@SiO_2_, FLAME). Five different types of PFCs were employed–PFCE, PFOB, FC-43, perfluorodichlorooctane (PFDCO), and 1,1,1-tris(perfluorotert-butoxymethyl)ethane (TPFBME) and to render multicolor fluorescence imaging capabilities, rhodamine B isothiocyanate, sulfo-cyanine 5, and fluorescein-4-isothiocyanate were covalently modified to silica shells [281]. Nanoprobes (PFCE@SiO_2_, TPFBME@SiO_2_, and FC-43@SiO_2_) enabled the triple-color ^19^F MR imaging in vivo for the first time.

#### 5.2.3. Carbon Based

**Figure 10:**
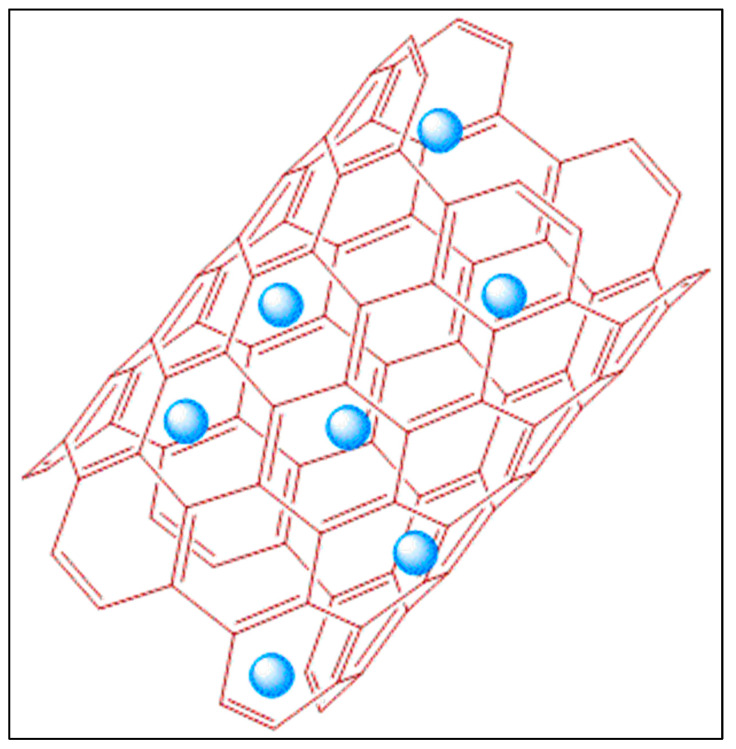
Carbon nanotubes with payload (blue circles).

Carbon nanotubes are nanoscale hollow cylindrical tubes consisting of rolled-up sheets of layer (single-walled or multi-walled) of graphene as sketched in Figure 10 [282]. The unique 1D structure, high mechanical tensile strength, high surface area, high thermal conductivity, chemical stability, effective resistance to any chemical impact, rich electronic polyaromatic structure, lightweight, possible surface functionalization, and the possibility to stuff the hollow interior with various imaging agents/drugs/molecules of interest renders them with manifold potential for applications [283,284].

Among the ROS, H_2_O_2_ plays an indispensable physiological role. Monitoring its pathological level gives valuable information on the multiple abnormalities occurring in the living entity. Fluorinated halloysite nanotube (HNT) was used to detect the low concentration of H_2_O_2_ by ^19^F NMR probe prepared using halloysite nanotubes ((Al_2_Si_2_O_5_(OH)_4_·nH_2_O)) and 3,5-bis(trifluoromethyl) benzeneboronic acid [285]. The halloysite nanotube with minor modifications uncovered its implementation as a fluorescent probe for selective and sensitive response to hyperoxide using 1-pyrenylboronic acid [286] in conjunction with a smart halloysite-based hydrogel prepared for H_2_O_2_-responsive drug delivery system [287].

### 5.3. Mixed/Hybrid NPs

To impart multifunctionality to an NP-based system, the convergence of organic and inorganic components is often pivotal, resulting in hybrid theranostic NPs. Ingenious and intelligent combinations of discrete functional nanostructured materials will enable the development of versatile nanomedical platforms for multimodal imaging or simultaneous diagnosis and therapy.

‘FETRIS’/Iron(III) tris-*β*-diketonate with PFPE were prepared with the idea that high-spin paramagnetic metal ions can profoundly alter the relaxation times *T*_1_ and *T*_2_ for the cell detection via ^19^F MRI [288]. The PFPE-based ligand–fluorinated β-diketones (FDK) was made using Claisen condensation between PFPE and p-methoxyacetophenone. Formed FDK was blended with an assortment of PFC derivatives (PFPE, PFPE diethylamide, PFOB, short PFPE oligomer perfluorotetraglyme), and the obtained blended oils were formulated into lipid-based paramagnetic nanoemulsions using microfluidization. Cytocompatible FETRIS agents were formed as FDK efficiently and irreversibly extracted Fe^3+^ ions from an aqueous solution into the fluorous phase in the PFPE-in-water nanoemulsions. The system had stretched out its application for intravenous injection and in vivo inflammation imaging [289]. To forge a multifunctional nanoprobe, Chen et al. used the strategy of the one-pot encapsulation method, where the PFCE was anchored through hydrophobic–hydrophobic interactions to Cu_1.75_S NPs and then trapped within the silica shell (Cu_1.75_S–^19^F@OFP–SiO_2_) [290]. Co-encapsulation agents were oleylamine-functionalized polysuccinimide and trimethoxy(octadecyl)silane. The resulting nanoprobes contained up to ~2.0 × 10^8^ fluorine atoms per particle with an ultrahigh ^19^F MRI signal.

Star polymers were contrived with a polyhedral oligomeric silsesquioxanes (POSS) core and partly fluorinated arms expecting the star’s arm to be visible by ^19^F MRI, while the POSS core to load drug molecules [291]. The arm of the star polymer consisted of TFEA and PEGMA monomers. The formation of eight partly fluorinated copolymer arms and a POSS core, with different sizes of NPs, molded depending on the length of the chain. The fabricated NPs were dissolved in the water, and the characterization showed a singlet for ^19^F NMR and augmented *T*_2_. Hybrid functionalized 2D carbon nanomaterials like graphene oxide and iron oxide (Fe_3_O_4_) NPs blended the magnetic characteristics of Fe_3_O_4_ and the photoluminescence of graphene oxide in MRI and fluorescence imaging [292]. Fluorinated graphite polymer was oxidized by strong oxidizing agents, to give highly fluorinated graphene oxide. Hybrid of fluorinated graphene oxide and iron oxide were prepared by co-precipitation technique using iron sulfate and iron chloride.

Another nanocomposite based on multifunctional Cu_7_S_4_−Au@PSI−^19^F/PEG was reported by Cui et al. [293]. Plasmonic nanostructures, including gold (Au) with a localized surface plasmon resonance (LSPR) peak in the transparent window (800−900 nm), had been recognized as a promising agent for photothermal therapy. LSPR in metals is due to free electrons whereas, for doped semiconductors, it is the cation vacancies (holes) that can be tampered with doping. Heterodimers (combining plasmonic nanostructures with chalcogenide) can aid in tuning the LSPR peak position. To attune the LSPR (~808 nm), the nanocomposite was prepared by growing a small Au domain on a heavily doped Cu_7_S_4_ to form a Cu_7_S_4_−Au heterodimer. Furthermore, by click chemistry, adding the fluorine component that had an inconsequential background, good sensitivity combined with high spatial resolution enhanced photothermal efficacy and decreased the optical damage to normal tissues.

A multifunctional hybrid vesicle was synthesized from PEGylated magnetite/PFOB–loaded organic/inorganic hybrid that could be used in dual-modality US/MR imaging and intensified image-guided high intensity focused ultrasound ablation [294]. The organic component was amphiphilic block copolymer–polystyrene-block-poly(acrylic acid). The hybrid shell layer was formed by shell cross-linking of the micelle of Fe_3_O_4_ NPs and PFOB using thiol-silane (3-mercaptopropyltrimethoxysilane). Hu et al. used copper sulfide (Cu_7_S_4_) coated with oleylamine functionalized 3,5-bis(trifluoromethyl)benzaldehyde fluorinated ligands for ^19^F MRI and photothermal ablation [295]. Ionic liquids (IL) based on 1-butyl-2,3-dimethyl-imidazolium (BMMI, BMMIBF_4_), 1-ethyl-3-methyl-imidazolium (EMI, EMIOTf), and 1-ethylpyridinium (EPy, EPyBF_4_) were explored with fluorinated anions, such as tetrafluoroborate (BF_4_) and trifluoromethanesulfonate (OTf) engendering a fluorinated IL-based activaTable ^19^F MRI platform (FILAMP) [80]. At 50 to 60 °C, these ILs confined inside the hollow mesoporous silica, and the pores sealed by stimuli-responsive copolymers could respond and release free IL upon biological stimulation.

A promising dual ^1^H/^19^F MRI probe was derived from lipid NPs, whose surface was modified with paramagnetic calcium-responsive Gd-chelates and encapsulated with PFCE [79]. The surfactants were dipalmitoylphosphatidylcholine with 5% of PEGylated phospholipid. Calcium helped in the contrast enhancement of ^1^H MRI. Water-soluble fluorinated NPs with metal-core and fluorinated ligands were reported by Arango et al. [116] with interesting MR features. A simple phase transfer method was used to bind gold NPs with fluorinating building blocks, and in vivo mice studies showed that they are suitable for ^19^F MRI/MRS. Magnetite (Fe_3_O_4_) NPs were modified with oleic acid, and this system formed self-assembled magneto-micelles with amphiphilic copolymers [296]. The fluorine-containing copolymers were synthesized using 2,2,3,4,4,4-hexafluorobutyl methacrylate (for holding hydrophobic drugs) and PEGMA (increased the hydrophilicity of the hybrid system). 5-fluorouracil was used to study the capability of the magneto-micelles to carry and load drugs and for in vitro release studies.

Some of the other hybrid systems include fluorinated polymer and manganese-layered double hydroxide NPs (Mn-LDH NPs) that benefitted the specific and sensitive detection of breast cancer [297]. Layered double hydroxides (LDHs) are 2D nanomaterials consisting mainly of divalent and trivalent cations in layers with anionic species intercalated between the layers. The cations could be metals, and the anions could be drug/polymers/siRNA, etc. pH-activated ^19^F MRI agents based on the PRE were specifically activated within the acidic tumour environment and were assembled with PFPE-based polymer and Mn-LDH NPs. Furthermore, Wang et al. reported the function of fluorine and nitrogen co-doped carbon dot complexed with Fe(III) put together as *T*_1_ CA in ^19^F MRI [298]. Using HeLa as a model cell line, the CA exhibited the lowest *T*_1_ relaxation, and in BALB/c mice, it displayed an accurate tumour image effect in addition to efficient renal clearance, low toxicity, high relativity, and bright luminescence.

A recent review by Mali et al. discusses recent advances in ^19^F-nanoparticles for ^19^F MRI [299], with a detailed analysis on the nanotechnologies employed for the design and applications of ^19^F-based nanoprobes. Table 4 gives information on most of the nanosystems discussed so far, including their preparation technique, fluorine component, characterization technique, their interesting pros and cons, and their applications.

## 6. Conclusions and Future Perspectives

Even though we see massive progress in the field of ^19^F MRI with fluorinated compounds, and in particular with PFCs, its clinical translation requires an in-depth understanding of their exact behavior from its intake until its complete degradation. PFCs are atypical molecules and overcoming specific barriers can shorten the distance to its clinical reality. Even though emulsion is one of the most used techniques to encapsulate PFC compounds, there are still various shortcomings that must be subjugated, including instability, heterogeneity, complex formulation procedure, split ^19^F signals, excessive retention of the agent within organs for months or longer are some of them [226]. The bottleneck attributes for PFC compounds are that they are immiscible with water or lipids, besides their organ retention and inefficacious chemical modification. It is simultaneously intriguing as well as exciting. These will open new approaches to drug delivery besides making scientists think differently. It is quintessential to comprehend the degradation of PFCs at extreme (pH/temperature/anoxic/hypoxic) conditions and the effects on high doses of PFCs as the knowledge of this is scarce considering PFCs are not naturally occurring compounds. Storage conditions need to be ascertained as long-term storage of PFC NPs remains a persisting setback.

Reviewing the recent works on nanosystems using fluorinated ligands and PFCs for ^19^F MRI, we see a prodigious stride in our understanding of formulation of the nanosystems and bringing novel ways of producing ^19^F signals. Not restricted to the fact that PFC is a unique liquid, the expedient means explored to encapsulate PFCs, above and beyond uncovering ways to produce molecules with a strong and single ^19^F signal, have been remarkable. The frequently confronted issues when attempting to make molecules equal to or better than PFCs is the complexity of the molecules’ preparation itself, in addition to the required number of synthetic steps, utilization of organic solvents, purification contingency, and reproducibility of the same. We can always see that the products that ultimately manage to pass to clinical stages are straightforward and, typically, would not require complex synthetic skills. One of the concerning issues is the usage of Gd^3+^ yet in these nanosystems, especially for making hybrid ^1^H/^19^F MRI. The unreserved fact that Gd has a plethora of advantageous properties cannot be denied. An effectual CA from another element can pave the way for unexplored possibilities and conceivably higher quality images.

The aerial perspectives for the future use of PFCs in an emulsion would be to experiment with the use of fluoro-surfactants instead of commonly used surfactants [302], to find a carrier system that has an affinity to PFCs and could hold PFCs inside with some interactions, to explore the therapeutic effect of PFCs, to make PFCs more hydrophilic by modifying them or to make them less hydrophobic so that they could be soluble in an organic solvent and to study the effect of using more than two PFCs concurrently. Similarly, one of the emerging and compelling nanosystems would be to combine different classes of NPs to produce a complementary system with a synergic effect. It is like a jigsaw puzzle where pieces are fit together to create a serendipitous combination.

The anticipated challenges faced when the chemical modification is performed, as already pointed out, is the usage of organic solvents, the increase in the number of reaction steps that decreases the overall yield of the final compound, which can hinder its progress to the clinic. Formulating a synthesis/preparation towards a ‘green’ approach can make its way to the clinic swifter. PFCs have some inimitable exclusive characteristics, and not only could this be utilized in imaging but also in other research areas like cell tracking, ^19^F-oximetry, inflammation probing, etc. Some of the PFC-based compounds are already FDA-approved for ultrasound-based CAs and it is only a matter of time before discovering the most befitting CA for ^19^F MRI, which would open a floodgate of applications.

## Figures and Tables

**Table 1:** Features of in vivo imaging modalities including their emission source, technique’s requirement of a contrast agent, their penetration depth, acquisition time and the targeted region for imaging.

Technique	Emission Source	Need of Contrast Agents	Spatial Resolution	Acquisition Time	Target
Optical Imaging	Visible and Near-Infrared Light	✓	millimeters (mm)	Seconds (S) to Minutes (Min)	Soft tissues
Photoacoustic (PAI)	Laser	✓	centimeters (cm)		Soft tissues
Ultrasound (US)	Sound Waves	✓	cm	S	Soft tissues
Computed Tomography (CT), Positron Emission Tomography (PET) Single-Photon Emission Computed Tomography (SPECT)	Gamma Rays	✓	mm	Min	Hard tissues and soft tissues
X-ray	X-rays	✓	micrometer (µm)	S	Hard tissues and soft tissues
Magnetic Resonance Imaging (MRI)	Radiofrequency Waves	✓	µm–mm	Seconds (S) to Hours	Deep soft tissue

**Table 2:** Comparative properties between hydrogen and fluorine.

Parameter	^1^H	^19^F
Natural abundance (%)	99.98	100
Spin	1/2	1/2
Gyromagnetic ratio (γ) in MHz/T	42.576	40.076
Relative sensitivity	1.0	0.834
Van de Waals’ radius (in Å)	1.2 (H–C)	1.35 (F–C)
The population ratio (N_E_/N_G_)	0.9999802	0.9999814
∆ϵ/kT at 3T	1.98 × 10^−5^	1.86 × 10^−5^
Lattice spacing	4.97 Å (Hydrocarbon)	5.9 Å (fluorocarbon)
Chemical shifts in ppm (NMR)	0 to 15	>350

**Table 3:** Survey of PFC molecules for potential MRI applications. The MF stands for the molecular formula/chemical formula, Mw is the molecular weight in g/mol, B.P is the boiling point, the density (D) is expressed in g/mL at 25 °C (lit.), the FNMR signals are estimated based on the molecular structure and based on the fluorine environment: S—Singlet, M—multiple peaks.

**Aromatic PFCs**	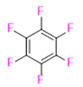 **Hexafluorobenzene (HFB)** ** MF = C_6_F_6_** ** Mw = 186.05 ** ** B.P = 80.2 °C ** ** FNMR signals = S **	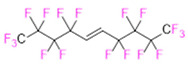 **Trans-1,2-bis(perfluoro-*N*-butyl)ethylene (TBPE)** **MF = C_10_H_2_F_18_** **Mw = 464.09** **B.P = 64,3 °C** **D = 1.675** **FNMR signals = M**	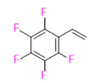 **2,3,4,5,6-Pentafluorostyrene (PFS)** ** MF = C_8_H_3_F_5_** ** Mw = 194.10 ** ** B.P = 139–140 °C ** ** D = 1.406 ** ** FNMR signals = 3 major peaks with spitting **
**Saturated Linear PFCs**	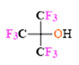 **Perfluoro-tert-butanol** ** (PFTB) ** ** MF = C_4_HF_9_O ** ** Mw = 236.04 ** ** B.P = 45.0 °C ** ** FNMR signals = S **	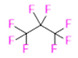 **Perfluoropropane** ** (PFP) ** ** MF = C_3_F_8_** ** Mw = 188.02 ** ** B.P = −36.6 °C ** ** FNMR signals = 2 major peaks with spitting **	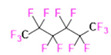 **Perfluorohexane (PFH)** ** MF = C_6_F_14_** ** Mw = 338.04 ** ** B.P = 56.6–57.2 °C ** ** FNMR signals = 3 major peaks with spitting **
	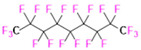 **Perfluorononane (PFN)** ** MF = C_9_F_20_** ** Mw = 488.06 ** ** B.P = ** ** 125–126 °C ** ** D = 1.799 ** ** FNMR signals = 3 major peaks with spitting **	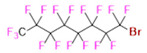 **Perfluorooctyl bromide (PFOB)** ** MF = C_8_BrF_17_** ** Mw = 498.96 ** ** B.P = 142 °C ** ** D = 1.93 ** ** FNMR signals = M **	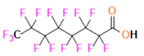 **Perfluorooctanoic acid (PFOA)** ** MF = C_8_HF_15_O_2_** ** Mw = 414.07 ** ** B.P = 189.0–192 °C ** ** D = 1.792 ** ** FNMR signals = M **
**Saturated Ring System PFCs**	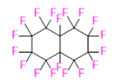 **Perfluorodecalin (PFD)** ** MF = C_10_F_18_** ** Mw = 462.08 ** ** B.P = 142 °C ** ** D = 1.908 ** ** FNMR signals = M **	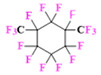 **Perfluoro-1,3-dimethylcyclohexane (PFDCH)** ** MF = C_8_F_16_** ** Mw = 400.06 ** ** B.P = ** ** 101–102 °C ** ** D = 1.828 ** ** FNMR signals = M **	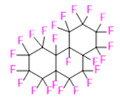 **Perfluoroperhydrophenanthrene (PFPHP)** ** MF = C_14_F_24_** ** Mw = 624.11 ** ** B.P = ** ** 212–218 °C ** ** D = 2.03 ** ** FNMR signals = M **
**Perfluoroethers and Polyethers**	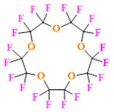 **Perfluoro-15-crown-5 ether (PFCE)** ** MF = C_10_F_20_O_5_** ** Mw = 580.07 ** ** B.P = ** ** 145 °C ** ** D = 1.780 ** ** FNMR signals = S **	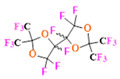 **Perfluoro-2,2,2’,2’-tetramethyl-4,4’-bis(1,3-dioxolane) (PTBD)** **MF = C_10_F_18_O_4_** **Mw = 526.08** **B.P = ~ 160 °C** **D = ~1.9** **FNMR signals = M**	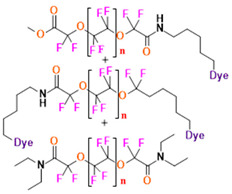 **Fluorescent** **‘blended’ PFPE Amides (FBPA)** **MF, Mw, B.P = Depends on repeat unit and the dye attached** **FNMR signals = 1 major peak, 4 minor peaks**
	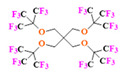 **Superfluorinated probe (PERFECTA)** ** MF = C_21_H_8_F_36_O_4_** ** Mw = 1008.23 ** ** FNMR signals = S **	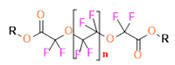 **Perfluoropolyether** ** (PFPE) ** ** MF, Mw, B.P = Depends on repeat unit and the R-group attached ** ** FNMR signals = 1 major peak and 1 minor peak **	
**Perfluoroamines**	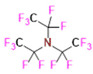 **Perfluorotriethylamine(PFTA)** **MF = C_6_F_15_N** **Mw = 371.05** **B.P = 68–69 °C** **D = 1.736** **FNMR signals = 2 major peaks with spitting**	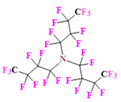 **Perfluorotributylamine** ** (FC-43) ** ** MF = C_12_F_27_N ** ** Mw = 671.09 ** ** B.P = 178.0 °C ** ** D = 1.884 ** ** FNMR signals = M **	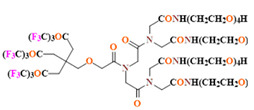 **^19^** **F Imaging Tracer (^19^FIT)** ** MF = C_63_H_94_F_27_N_7_O_27_** ** Mw = 1894.41 ** ** FNMR signals = S **
**Perflurosilanes**	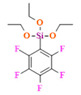 **(Pentafluorophenyl)triethoxysilane (PFPTS)** ** MF = C_12_H_15_F_5_O_3_Si ** ** Mw = 330.32 ** ** B.P = 69 °C ** ** D = 1.242 ** ** FNMR signals = 3 major peaks with spitting **	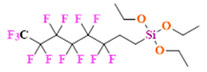 **^1^** **H,^1^H,^2^H,^2^H-Perfluorooctyltriethoxysilane (PFOTS)** ** MF = C_14_H_19_F_13_O_3_Si ** ** Mw = 510.36 ** ** B.P = 220 °C ** ** D = 1.329 ** ** FNMR signals = M **	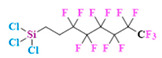 **Trichloro(^1^H,^1^H,^2^H,^2^H-perfluorooctyl)silane (TCPFOS)** ** MF = C_8_H_4_C_l3_F_13_Si ** ** Mw = 481.54 ** ** B.P = 192 °C ** ** D = 1.30 ** ** FNMR signals = M **

**Table 4:** The selected examples of studied fluorinated and PFC nanosystems explaining their preparation technique, fluorine component, characterisation techniques used for studying various aspects of the nanosystems, pros and cons based on the synthesis/preparation and the practicality, and applications. * The abbreviations are expanded at the ‘Abbreviation’ session.

Type	Name	Preparation Technique	Fluorine Component	Characterisation *	Pros and Cons	Application	Ref
**POLYMERIC**	Fluorous colloidal NPs	Copolymer by ATRP. NPs formation by self-assembly to micelle	Trifluoroethyl methacrylate	DLS (260 nm), TEM, FMRI, FC, CM, UV-Vis, CyA–on macrophage cells, animal studies–female athymic NCR nude mice for breast cancer	Simple preparation of copolymer	Immune cell tracking and systemic disease monitoring	[187]
					No surfactant		
					Little off target accumulation		
					Tumour-homing		
	Poly(OEGA-*co*-TFEA)-*b*-poly(*St*-co-VBA)	Polymerisation by RAFT and NPs by PISA	2,2,2-trifluoroethyl acrylate	FMRI and NMR, DLS, TEM, CM	Little or no cytotoxicity–Chinese Hamster Ovarian cells	In vivo cell tracking	[190]
					Multiple NPs morphologies by controlling reaction time and polymer chain length in one preparation (spherical, worm, vesicle)		
	ROS-responsive fluorinated polymers	Polymer by ATRP and NPs by self-assembly	2,2,2-trifluoroethyl methacrylate	H and F NMR, FMRI, DLS (62, 32 and 18 nm), UV-Vis	Enhanced sensitivity for acidic microenvironment and the presence of ROS	ROS/pH dual-responsive ^19^F MRI agent	[191]
					The concentration of H_2_O_2_ studied (~1 M) were higher than biological levels (50–100 μM)		
					6-step synthesis that requires purification		
					“OFF–ON” regulation of NPs to acidic environment		
	Amino activable nanoprobe- p(mPEGMA)-*co*-poly(AMA-DNBS-F) (PEDF nanoprobe)	Copolymers by RAFT polymerisation and nanoprobe by nanoprecipitation	Trifluoromethyl-containing segments	H and F NMR, DLS (33 nm), FMRI, TEM, FTIR, CLSM, in vivo imaging in tumours–xenograft tumour models in mice	2 step preparations for monomers	In vivo bio-thiols imaging	[192]
					Highly sensitive to bio-thiols		
					Water soluble		
	Fluorinated block copolymers NPs	RAFT for the block polymers and NPs by self-assembly in aqueous solution	2,2,2-trifluoroethylamide L-arginine methacrylamide	H, F- NMR, DLS (25 to 60 nm), TEM	Fluorinated functionalities in the hydrophilic shell	MRI Imaging	[300]
					Increased *T*_2_		
	^19^F MRI-detectable drug delivery system	Layer-by-layer technique deposition of polyelectrolyte shells on nanoemulsion drops	Polyelectrolyte Nafion–fluorinated anionic polymer	DLS (170 nm), LDV, NTA, C-SEM, QCM, FMRI	Sufficient SNR ratio	Passive tumour targeting and drug delivery	[193]
					Highly cationic particle (+68 ± 5 mV)		
	Self-assembled ^19^F nanoprobes	Self-assembly of amphiphilic redox-responsive ^19^F-containing polymers and NIR-absorbing ICG molecules	3,5-Bis(trifluoromethyl) benzoic acid part in the polymer	TEM, DLS (40 nm), UV–Vis, FNMR and MRI, TEM	Water-soluble	Accurate sensing and imaging of tumours	[66]
					In vivo and in vitro studies–HepG2 tumour-bearing cells and mice		
					High SNR ratio		
					Good biocompatibility		
					5 steps for preparation with purification requirement and moderate yield		
					Novel system which has potential to be extended for imaging other tumour targets		
	Multi-functional fluorocarbon NPs	Single and double emulsion	PFD, PFH, perfluorooctane, PFOB, PFCE	DLS (200 nm–200 µm), SEM, CM, FC, FI, FMRI, Cell viability–primary human dendritic cells, histology	Customizable NPs, minimal toxicity	In vivo imaging and targeting applications	[194]
					Size smaller than 200 nm is not formed by this NP formation		
	PLGA PFPE	Emulsification (Sonicator)–1:1 molar ratio of autoclaved PFPE and sterile filtered Pluronic	PFPE	DLS (103 nm), FNMR and MRI, FM, cellular viability–diabetogenic mice T cells	Specificity for the labelled cells	Non-invasive monitoring the trafficking of cellular therapeutics	[195]
					Reliable estimates of the apparent number of cells from image data		
	PFCE encapsulated PLGA	Single emulsion	PFCE	DLS, FNMR and MRI, SANS, animal studies–male Wistar rats, mouse, mice, cell studie–primary murine/human dendritic cells	Biocompatible NPs	US and ^19^F MRI	[197]
					Better acquisition time	Murine cardiac ^19^F MRI/MRS	[199]
					Obtains complimentary information when in combination with other imaging agents	In vivo PAI, ^19^F MRI and fluorescent imaging (FI)	[198]
					NPs loaded with chemotherapeutic drugs could give it a theranostic effect, Resomer RG 502 H, lactide: glycolide molar ratio 48:52 to 52:48 is the mostly used PLGA. The other ratios of lactide: glycolide and also their end group might give interesting results. The encapsulation efficiency of PFC could be studied each time to better understand the sensitivity	FMRI and CT (with gold NPs)	[200]
						SPECT/PET and ^19^F MRI	[204]

	Chitosan coated PLGA -PFOB NPs	Single emulsion by homogenisation followed by sonication using 1.5% sodium cholate	PFOB	DLS (170 nm), CLSM, FC, FNMR and FMRI, TEM	Background-free signal compared to Gd (III) and super paramagnetic iron oxides NPs	Labelling and tracking therapeutic cells in vivo	[206]
					As chitosan coating is just a physical adsorption, the stability of it has to be verified in biological environment		
					Size of NPs is increased (200–400 nm) after the chitosan coating		
	PEGylated PLGA NPs (PLGA NP (NIR700 + PFC)-PEG-800 CW	O/W emulsion and solvent evaporation-extraction method	PFCE	DLS (240–250 nm), TEM, FMRI, TEM, FM, histology, cell culture–murine breast carcinoma cell line	Quantitative 3D information from deeper tissues	In vivo imaging	[212]
					Rapid qualitative optical monitoring		
	PLGA–PEG folate-receptor-targeted NPs	Single emulsion-evaporation (1.5% sodium cholate surfactant)	PFOB	DLS (150 nm), FC, CLSM, F MRI, NIRS, CyA-KB cells	Encapsulate imaging agent and drug	Theranostic NP	[207]
					Insufficient SNR in vivo for FMRI		
					The loading capacity of the NPs is low for doxorubicin and ICG (0.04% and 0.127%)		
	Doxorubicin-conjugated PFPE NPs	Polymers by RAFT polymerization	PFPE	DLS (8.1, 9.3 and 8.3), FNMR, MD	Improved cellular uptake	Improved therapeutic efficacy	[222]
					Deep tumour penetration		
					Studies done using 3D tumour spheroids		
	F_3_-PLGA and F_9_-PLGA	Nanoprecipitation–surfactant free	Fluorinated PLGA (2,2,2-trifluoroethanolamine, nonafluoro-t-butoxyethylamine)	DLS (~54 nm and 58 nm), TEM, F NMR, FM, CyA–immortalized human glomerular endothelial cells and podocytes	No surfactant used	Theranostic NPs	[217]
					Encapsulate hydrophobic drugs		
					The reaction yield of the fluorinated polymer is not understood		
**HYPERBRANCHED**	Multifunctional hyperbranched polymers containing ^19^F	RAFT polymerization for polymer, NPs by self-assembly in water	2,2,2-trifluoroethylacrylate	DLS (∼13 nm), GPC, TEM, FNMR and MRI, CT	Direct dissolution in water	Quantitative ^19^F MRI CA	[232]
					Biodegradable		
					3 step preparation and the final product is not pure (3 mixture products)		
					FNMR with multiple peaks		
					*T*_2_ shortened		
	PFPE based hyperbranched NPs conjugated with targeting aptamers	RAFT polymerization–for NPs, click chemistry for aptamers attaching	PFPE	F-DOSY (<10 nm), FM, FC, CrM, MD, FNMR and MRI	Superior MR imaging sensitivity and fluorine content -breast cancer cells	Quantitative ^19^F MRI CA	[233]
					Low-cost fluorescence imaging		
					Unsuitable for long term studies due to faster clearance from the body		
					Accumulation of polymer in the liver was observed after 48 h and the ^19^F signal could be still detected in the liver		
	Fluorinated hyperbranched polyether copolymers	ROMBP and copolymerization for polymers and self-assembly of the colloids	2-[(2,2,2-trifluoroethoxy) methyl]oxirane/epifluorohydrin	DLS (160–200 nm), H NMR and F MRI, FM, HPLC, cytotoxicity studies - immortalized human glomerular endothelial cells and immortalized human podocytes	Repair damaged kidney glomerular cells in vitro	New generation ^19^F MRI nanotheranostics	[234]
					Negligible cytotoxicity		
					Narrow size distribution		
					Relatively long *T*_1_		
					Higher amount of F gives less SNR		
**DENDRIMERS**	Fluorinated Gd(III)-DOTA complexes	Convergent synthesis for polymer and self-assembly for NPs	Fluorinated amino acid group	F NMR, DOSY, H and F MRI, KB cells for in vitro cytotoxicity study, animal imaging—Sprague Dawley female rats	Substantial improvement in relaxation rate and SNR ratio	CA for high field imaging	[239]
					Easily cleared through the kidneys		
					The fluorine in the surface layer of dendrimers is toxic which can be diminished by burying the fluorine further into the dendrimer interior		
	Second-generation dendron	Sonogashira coupling, alkyne deprotection and CuAAC	PFTB group attached to the dendron	FNMR	Higher number of equivalent fluorine than commercially available ^19^F MRI probes	Probes for ^19^F MRI	[240]
					Too unpolar to be water-soluble		
					Just one characterisation technique used		
	Pseudo-symmetrical fluorines dendrimers	Polymer prep–bromination and Williamson ether synthesis, NPs by self-assembly	Bis(4-fluorophenyl) trifluoromethyl carbinol group	FNMR and MRI	Large amount of fluorine with a single NMR peak	^19^F MRI-guided drug therapy	[241]
					Optimize ^19^F relaxation time		
					High sensitivity		
					Reliable quantification		
					Comparatively low yield (8%) for 11 synthesis steps		
	Self-assembled fluorinated amphiphiles	Convergent way–Sonogashira coupling and Williamson ether synthesis for polymer, NPs by self-assembly	Fluorinated benzyl group	FNMR, DLS (6.3 nm), TEM	Quantifying drugs, detecting drug microenvironments and weak interactions	^19^F NMR/MRI guided drug therapy.	[242]
					Several synthetic step for the preparation with most of them requiring separation		
**NANOHYDROGELS**	Chitosan	Ionic gelation using hyaluronic acid and tripolyphosphate	4,4,4-trifluorobutyric acid	DLS (274 nm), ELS (+30 mV), FNMR (−66 ppm), HNMR, TGA, DOSY, IR	Good biocompatibility toward murine macrophages cell line	Chitosan drug delivery systems for MRI lymphography	[247]
					Degree of substitution is comparatively low (0.3% and 20%) and varies between different substitutes, and determination is laborious		
	Diblock polymers	Self-assembly by heating in aqueous solution	Poly[*N*(2,2 difluoroethyl)acrylamide]	SLS (100 and 67 nm), TEM, C-TEM, FNMR	Good sensitivity	^19^F MR imaging–angiogenesis imaging or the labelling of pancreatic islets	[248]
					Non-cytotoxic for several cell lines		
					Long synthesis steps for preparation of polymers		
	Fluorinated amphiphilic polymers	Self-assembly of polymers–direct dissolution of amphiphilic polymers in PBS buffer	-CF_3_ groups attached to the chains of polymer	DLS (6- 14 nm), FNMR, FMRI–phantom and animal imaging, CM, CyA-HeLa cells	Enhancement in *T*_2_ relaxation times by increasing the segment mobility	Multimodal imaging and therapeutic applications.	[249]
	Superhydrophilic ^19^F MRI CA	Hydrogel matrix attached to zwitterionic, fluorinated and alkynyl molecule by click chemistry	The fluorine atoms on trifluoromethyl groups	HMRS, FTIR, GPC, CD, Rheometer, SEM, FMRI, degradation study–female BALB/c mice, CyA-Dendritic cells, NIH 3T3 cells	Gelation properties of hydrogels unaffected by labelling CA	Real-time FMRI to precisely locate and quantify the degradation rate of hydrogel scaffolds in vivo	[250]
					3D-stereoscopic and 2D-anatomical information		
**LIPIDS**	Antigen-loaded PFC particles	High-frequency mixing of the liquid PFC with a cationic lipid mixture-particles coat with PEG	PFH or PFCE	CM, TEM, F NMR, Cytotoxicity in transplanted pancreatic islets and beta cell-like cells and T-cell proliferation assay	Improving pancreatic islets transplantation technique	Theranostic PFC NPs	[255,256]
					Good cell viability and no change in cells’ phenotypical properties		
					High resolution localization of transplanted cells		
					The use of PFCE is better than PFH because the latter have 3 peaks in FNMR which reduces its sensitivity		
	Thermally responsive lipid nano-emulsion	Nano-emulsion	Modified α-tocopherol	FNMR, DLS (50 nm), ZP	Proved that *T*_2_ changes more than *T*_1_ due to variation in temperature for FNMR	Potential tumour diagnosis	[258]
					The temperature studied is extreme (37 and 42 °C) compared to real tumour		
	Multifunctional paramagnetic PFC NP	Microfluidization	PFCE	DLS (132 nm), AFM, UV–vis, FM, cellular toxicity on bronchial epithelium, FC, clinical pathology, FMRI	Enhanced intratumoural penetration	PFC NP delivery from intravenous applications to intratracheal use (for lung cancer)	[259]
					NPs stored under very special condition		
					The lipid surfactant used have a laborious preparation		
					The studied NPs contain Gd^3+^ as Gd-lipid chelates		
**MICELLE**	Fluorinated thermoresponsive assembled protein (F-TRAP)	Self-assembly micelle	Fluorinated amino acids within a protein (5,5,5-*DL*-trifluoroleucines)	DLS (30 nm), FA, SLS, CD, MALDI-TOF-MS, TEM, turbidometry, FNMR, FMRI, Animal studies-mouse xenograft model of human breast cancer	No change in *T*_1_	Thermoresponsive ^19^F MRI/MRS-traceable theranostic agents	[260]
					Doxorubicin encapsulation and thermoresponsive release		
					Zero echo time ^19^F MRI was used to get the direct imaging of protein as after micelle formation, there is a reduction in *T*_2_		
					The release of drug is at 45 °C (usually tumour temperature range is 37 °C to 39 °C)		
**INORGANIC**	Gold NPs protected by fluorinated ligands (F- MPC)	Homogeneous phase synthesis	Fluorinated tetraethylene glycol part of the ligand	DLS (10 nm), TEM, HAADF-STEM, FNMR, UV-Vis, ESR, CLSM, cell interaction with HeLa cells	Elimination of the use of surfactants	Nanovector	[266]
					Size may help to reach small vasculature vessels		
					Soluble in many organic solvents		
					The preparation of fluorinated ligands contains 6 steps, most of them requiring purification		
	Functionalized gold NPs	Homogeneous phase synthesis	Fluorinated tetraethylene glycol part of the ligand	DLS, HNMR, TEM (1.5–2 nm), UV-Vis, FNMR and MRI	Water-soluble	Dual ^1^H/^19^F MRI	[267]
					Good quality MRI images		
					Same as ref [266] + Gd(III) is embedded deep in the layer of Au NPs that causes reduction in *T*_1_ relaxation times of bulk water proton		
	Gold NP functionalised with fluorine atoms	Reduction of HAuCl_4_ in the presence of NaBH_4_	PFTB	ICP-MS, F-NMR/MRI, UV-Vis, TEM, Cell viability and apoptosis assays -MDA-MB-231, C33-A and MDA-MB-435S cell lines, MTS CyA	Colloidal stability in water and other solvents	^19^F MR imaging	[268]
					Single chemical shift		
					Long storage		
					High fluorine loading		
					Long preparation and purification procedure for the fluorine ligands		
					The position of fluorine in the NPs is not established		
	Hollow mesoporous silica NPs (HMSN-PFCE)	Modified protocol from [301]	PFCE	DLS, SEM (290 nm), TEM, MRI, NMR, PAGE	Prolonged circulation time	Dual MRI (^1^H and ^19^F)	[270]
					Helps in understanding the effect of loading agent on the biodistribution of NPs		
					Better biodistribution of NPs		
					The study is majorly applicable to systems whose cargo is on the outer surface		
	Fluorinated mesoporous silica NPs (FMSNs and polyFMSNs)	Repeated impregnation-calcination process	Fluorosilane or polyfluorosiloxane	TGA, TEM (140 nm), DLS, FNMR and MRI, XPS, relaxometric properties	Colloidal stability	Dual MRI (^1^H and ^19^F)	[273]
					Increase in ^19^F relaxivities		
					Meticulous NPs preparation		
					Contains Gd^3+^		
					The detection of probe might be impeded by the strong reduction of *T*_2_ after NPs formation		
	PEG modified silica NP	Dehydration polymerizing reaction–PFCE including micelle as a platform	PFCE	TEM, DLS (50 nm), ^1^H/^19^F MRI	High sensitivity	Tumour imaging	[274]
					Water stability		
					Information on long term stability, encapsulation efficiency of PFCE is deficient		
	Silica multifunctional core–shell NPs (FLAMEs)	PFCE-phospholipid nanoemulsion by sol-gel process using a novel surfactant, PAP	PFCE	DMS (76 nm), F NMR and MRI, TEM, biocompatibility by MTT assay-colon-26 cells, Passive targeting, and accumulation- mice bearing a tumour	High sensitivity	Detection of gene expression and in vivo tumour imaging	[277]
					Modifiability of the surface, biocompatibility		
					In vivo stability		
					The FLAME NPs needs to be PEGylated as naked NPs is trapped immediately by the RES		
					The information on long term stability of NPs is lacking		
	Mesoporous FLAME (mFLAME)	PFCE emulsion by Sol–gel process	PFCE	DLS (165 nm), TEM, FNMR and MRI, CLSM, FC, MTT CyA-KB cells, FM	Ample cellular uptake and drug release in folate receptor-overexpressing tumour cells	Theranostic cancer treatment	[278]
					Drug release abilities at lower pH. (pH 5)		
					Efficient tumour cell internalization		
	Gd^3+^ complexes on FLAME NPs surface (FLAME-SS-Gd^3+^)	Gd^3+^ complexes were attached to the FLAME surface by disulfide linkers	PFCE	DLS (53.4 nm), FNMR and MRI, ICP-AES	Smart nanoprobe–based on PRE effect	Novel ^19^F MRI probes that visualize reducing environments	[279]
					In vivo imaging		
					High SNR ratio		
	PFC based ^19^F MRI nanoprobes (PFC@SiO_2_, FLAME)	PFC emulsion by sol–gel process	PFCE, PFOB, FC-43, PFN, PFDCO, TPFBME	DLS, TEM (40–120 nm), FI-RAW264.7 cells, H MRI and FMRI, hepatic uptake in mouse	*T*_2_ values -relatively longer than polymer-based or inorganic ^19^F MRI nanoprobes	Multicolour MRI probes	[281]
					In vivo triple-colour ^19^F MRI		
					The shelf-life information is lacking for the NPs		
	Fluorinated paramagnetic CAs	Multistep synthesis–cycloaddition reaction	Nonafluorinated carboxylic acid	FNMR, relaxivity measurements, MD	Relaxation times depending on the lanthanide ion	^19^F MRI	[35]
					Low solubility in aqueous media		
	Hexagonal-phase NaGdF_4_:Yb^3+^/Tm^3+^ NPs	Hydrothermal method	NH_4_F/NaF	XRD, SEM, EDX, UV, photoluminescence spectra, EPR	Conducive to the UV light	IR tomography and MRI	[261]
					Good water solubility		
					Lanthanide-based upconversion NPs		
	Inorganic nanocrystals-PEG-coated CaF_2_ nanocrystals	Solvothermal approach	CaF_2_	H and C and F-NMR, DLS (<10 nm), TEM, XRD, EDX, FTIR, TGA, mouse model of inflammation	Maximal ^19^F density	Imaging tracers for in vivo ^19^F MRI	[263]
					Average out homonuclear dipolar interactions		
					Direct and real-time in vivo ^19^F MRI		
					Chemically surface modifiable		
					Long *T*_2_		
	Halloysite nanotubes- benzeneboronic acids (HNTs-6FBB)	One-pot synthesis	3,5-bis(trifluoromethyl) benzeneboronic acid	FNMR (−60 ppm), XRD, FTIR, XPS, TEM, EA (0.31% F)	Relatively long *T*_2_	Selective response toward H_2_O_2_	[285]
					Water dispersibility		
					Detection of H_2_O_2_ is based on a very minute shift in FNMR (0.2 ppm)		
					Low cell cytotoxicity		
**MIXED/HYBRID**	Fe(III) tris-β-diketonate with PFPE (‘FETRIS’)	Microfluidization –metal-binding β-diketones conjugated to PFPE using pluronic surfactant	PFPE and PFPE derivatives, PFOB	DLS (140 nm to 200 nm), FNMR and FMRI, cell labelling-rodent glioma cell line	Ability to tune *T*_1_ by Fe concentration	In vivo detection of cell therapies and inflammatory cells	[288]
					Low cytotoxicity		
					Small rates of metal leakage in the presence of EDTA in vitro and after cell labelling		
	Cu_1.75_S–^19^F@OFP–SiO_2_	One-pot encapsulation method-PFCE anchored to Cu_1.75_S NPs and trapped within the silica shell	PFCE	DLS (20.8 nm), TEM, FMRI, PTT	Ultrahigh F signal	Ablation and sensitive multimodal imaging	[290]
					Biocompatible		
					Capable of both in vivo imaging (F-MRI) and photothermal ablation		
					Presence of excess of metals in a single probe!		
					The degradation of this complex should be evaluated since without the SiO_2_ coating it is cytotoxic		
	Fluorinated POSS-star polymers	Synthesis of star polymers by RAFT polymerization and polymer formation in water	2,2,2-Trifluoroethyl acrylate in the ligands attached to POSS	DLS (8–10 nm), FNMR and FMRI	High imaging intensity	Theranostic agents for cancer diagnosis and treatment	[291]
					No surfactants		
					The yield for the formation of star polymers is low and extreme conditions for preparation		
	Hybrid of fluorinated graphene oxide and iron oxide (IFGO)	Graphene oxide-Hummer’s method. Hybrid–co-precipitation	Fluorinated graphene	DLS (8–10 nm), FMRI, XRD, XPS, SEM and HRTEM, FTIR, MTT CyA-benign breast epithelial cell line, Raman, UV-Vis, hysteresis	Additional imaging modality–magnetic targeted drug delivery	Superior CAs for MRI and fluorescent imaging	[292]
					Increased magnetic saturation-better contrast		
	Cu_7_S_4_−Au heterodimer Cu_7_S_4_−Au@PSI−^19^F/PEG nanocomposites	Wet-chemical method for Cu_7_S_4_-Au nano seeds followed by click chemistry	2,2,2-trifluoro-*N*-2-propyn-1-yl-acetamide	DLS, HRTEM (27 nm), XRD, EDX, HAADF-STEM, XPS, STEM, F NMR and MRI, CT, cell viability-4T1 cell lines, PTT–liver of female mice	Deep penetration	Multimodal imaging guided photothermal therapy	[293]
					High spatial resolution		
					Enhances the photothermal efficacy		
					Long preparation for the nanocomposite		
	Mn-LDH@PFPE NPs	Composite system by conjugating a PFPE onto the surface of manganese-incorporated layered double hydroxide	PFPE	NMR and MRI, DLS (10 nm), TEM, GPC, CM, MTT assay–MDA-MB- 468 breast cancer cells, histopathologic examination	High specificity to breast cancer cells	Potential “smart” ^19^F MRI agent for detection of cancer diseases	[297]
	Fe^3+^@F,N-CD (fluorine and nitrogen co-doped carbon dot)	Simple microwave-assisted thermal decomposition method–from glucose and levofloxacin	Levofloxacin	DLS (16 nm), TEM, GPC, FTIR, XPS, FM, ESR, cytotoxic studies–HeLa cells, In vivo experiments -4T1 tumour bearing BALB/c mice, FMRI, CLSM	High *T*_1_ relaxivity	*T*_1_-weighted MRI CA	[298]
					Strong photoluminescence		
					Low synthetic cost		
					Low toxicity		
					Cannot be used for long term imaging in the body as they are excreted in a very short time from the body		

## Data Availability

Not applicable.

## Abbreviations

^19^F MRI/FMRI	Fluorine-19 Magnetic Resonance Imaging
^19^F NMR	Fluorine-19 Nuclear Magnetic Resonance
^1^H MRI/HMRI	Proton Magnetic Resonance Imaging
AFM	Atomic Force Microscopy
ATRP	Atom Transfer Radical Polymerization
BALB/c	Albino, laboratory-bred strain of the house mouse
BODIPy	Boron-dipyrromethene
C NMR	Carbon-13 Nuclear Magnetic Resonance
CA	Contrast Agent
CD	Circular Dichroism Spectroscopy
CLSM	Confocal Laser Scanning Microscopy
CM	Confocal Microscopy
CrM	Correlation Microscopy
C-SEM	Scanning Cryo-Electron microscopy
CT	Computed Tomography
C-TEM	Cryo-Transmission Electron Microscopy
CuAAC	Copper-Catalyzed Azide-Alkyne Cycloaddition
CyA	Cytotoxicity Assays. The usually used assays are I. 3-(4,5-dimethylthiazol-2-yl)-2,5-diphenyl tetrazolium bromide assay (MTT Assay), II. Lactate Dehydrogenase colorimetric Colorimetric assay Assay (LDH),
DC	Dendritic cellsCells
DLS	Dynamic Light Scattering
DOSY	Diffusion Ordered 2D-NMR Spectroscopy
EA	Elemental Analyzer
EDX	Energy-Dispersive X-Ray Spectroscopy
EGDMA	Ethylene Glycol Dimethylacrylate
ELS	Electrophoretic Light Scattering
EMA	European Medicines Agency
EPR	Electron Paramagnetic Resonance Spectrum
FA	Fluorescence Anisotropy
FC	Flow Cytometry
FDA	The Food and Drug Administration
FDK	Fluorinated β-diketones
Fe_3_O_4_	Iron Oxide/Magnetite
FI	Fluorescence Imaging
FITC	Fluorescein Isothiocyanate
FLAME	Fluorine Accumulated Silica NP for MRI Contrast Enhancement
FM	Fluorescence Microscopy
FTIR	Fourier Transform Infrared Spectroscopy
GBCAs	Gadolinium Based Contrast Agents
Gd(III)/Gd^3+^	Gadolinium-III
GPC	Gel Permeation Chromatography
HAADF-STEM	High-Angle Annular Dark-Field Scanning Transmission Electron Microscopy
HBIPF	Hyperbranched Iodopolymer Containing ^19^F
HepG2 Cells	Hepatocellular carcinoma cells
HNMR	Proton Nuclear Magnetic Resonance
HNT	Halloysite Nanotube
HPLC	High-Performance Liquid Chromatography
HRTEM	High Resolution Transmission Electron Microscopy
ICG	Indocyanine Green
ICP-AES	Inductively Coupled Plasma Atomic Emission Spectrometry
ICP-MS	Inductively Coupled Plasma Mass Spectrometry
KB cells	Human Nasopharyngeal Epidermal Carcinoma
LDV	Laser Doppler Velocimetry
LSPR	Localized Surface Plasmon Resonance
MALDI-TOF-MS	Matrix-Assisted Laser Desorption Ionization-Time-Of-Flight Mass Spectrometry
MD	Atomistic Molecular Dynamic Simulations
mPEG	Monodisperse Poly (ethylene-glycol)
MR	Magnetic Resonance
MRI	Magnetic Resonance Imaging
MRS	Magnetic Resonance Spectroscopy
NIR	Near Infrared
NIRS	Near Infrared Spectroscopy and Imaging
NMR	Nuclear Magnetic Resonance
NP	Nanoparticle
NTA	Nanoparticle Tracking Analysis
OEGA	Oligo(Ethylene Glycol) Methyl Ether Acrylate
PAGE	Polyacrylamide Gel Electrophoresis
PAI	Photoacoustic Imaging
PEG	Poly (ethylene-glycol)
PEGMA	Poly-(ethylene glycol) methyl ether methacrylate
PET	Positron Emission Tomography
PFC	Perfluorocarbon
PFCE	Perfluoro-15-crown-5 ether
PFDCO	Perfluorodichlorooctane
PFOB	Perfluorooctyl Bromide
PFP	Perfluoropropane
PFPE	Perfluoropolyether
PFTB	Perfluoro-tert-butanol
PLGA	Poly (lactic-*co*-glycolic acid)
PRE	Paramagnetic Relaxation Enhancement
PTT	Photothermal Therapy
QCM	Quartz Crystal Microbalance
RAFT	Reversible Addition−Fragmentation Chain-Transfer Polymerization
RF	Radiofrequency
ROMBP	Ring-Opening Multibranching Polymerization
ROS	Reactive Oxygen Species
SANS	Small Angle Neutron Scattering
SEC	Size Exclusion Chromatography
SEM	Scanning Electron Microscope
SLS	Static Light Scattering
SNR	Signal/Contrast-to-Noise Ratio
SPECT	Single-Photon Emission Computed Tomography
STEM	Scanning Transmission Electron Microscope
TAM	Tumour-associated macrophages
TEM	Transmission Electron Microscope
TFEA	2,2,2-trifluoroethyl Acrylate
TGA	Thermogravimetric Analysis
TM	Turbidometry
TPFBME	1,1,1-tris(perfluorotert- butoxymethyl)ethane
UC	Upconversion
US	Ultrasound
UV–Vis	UV/Vis Absorption Spectra
XPS	X-Ray Photoelectron Spectroscopy
XRD	X-Ray Diffraction
ZP	Zeta Potential
